# D-dimer and lower limb ultrasound as prognostic factors for recurrent deep venous thrombosis and pulmonary embolism: A systematic review and meta-analysis

**DOI:** 10.1371/journal.pone.0340158

**Published:** 2026-05-15

**Authors:** Sebastián Salinas-Mendoza, Álvaro Javier Burgos Cárdenas, Sugeich del Mar Meléndez Rhenals

**Affiliations:** 1 Department of Internal Medicine, Universidad Nacional de Colombia, Bogotá D.C, Colombia; 2 Department of Internal Medicine, Hospital Serena del Mar, Cartagena, Colombia; 3 Subdirección de investigaciones Fundación Cardioinfantil, Instituto de Cardiología, Bogotá D.C, Colombia; Università degli Studi di Milano, ITALY

## Abstract

Venous thromboembolic disease is a chronic, recurrent condition. The optimal duration of anticoagulation therapy remains uncertain. We aim to evaluate D-dimer and lower limb ultrasonography as prognostic tools for the recurrence of venous thromboembolism. A search was conducted on May 28, 2022, in the Medline, Embase, and Cochrane databases. Inclusion criteria encompassed cohort studies, case-control studies, and clinical trials. Two reviewers independently screened all records and analyzed studies for inclusion/exclusion criteria, as well as risk of bias, using a structured framework (PROSPERO ID: CRD42022341082). The initial search yielded 4652 titles and abstracts. After removing 777 duplicates and reviewing 3875 titles and abstracts, 48 articles providing information on D-dimer and/or lower limb ultrasonography as prognostic factors were finally included. Very low-certainty evidence suggests that both residual vein thrombosis (RVT) at anticoagulation discontinuation and a positive post-treatment D-dimer are significantly associated with an increased risk of recurrent venous thromboembolism (VTE). For RVT, the pooled analysis showed a two-fold increase in risk (OR 2.00, 95% CI 1.02 to 3.94; I² = 85.7) while for positive D-dimer, the risk was similarly elevated (OR, 2.48; 95% CI, 1.85–3.33; I² = 56.0). In conclusion, very low-quality evidence suggests that abnormal D-dimer and RVT are associated with recurrent VTE; however, this association is inconsistent due to significant heterogeneity and wide prediction intervals. These biomarkers should be interpreted with caution as isolated predictors in clinical practice.

## Introduction

Venous thromboembolism (VTE) encompasses pulmonary embolism (PE) and deep vein thrombosis (DVT). VTE has an estimated incidence of 0.5 to 2 per 1000 people per year within the general population [1,2]. This represents a significant global burden; after acute coronary syndrome and stroke, it is the third most common cause of vascular death [3].

VTE is a chronic disease with a strong tendency to recur. It generally recurs after treatment discontinuation, and it can also recur in the presence of adequate therapy in a non-negligible proportion of patients. The risk of recurrence after an episode of venous thromboembolism (VTE) triggered by transient risk factors, such as hospitalization or surgery, is lower than in patients with persistent risk factors, like cancer, inflammatory bowel disease, or antiphospholipid syndrome. Patients with VTE in the absence of any identifiable risk factors, known as unprovoked venous thromboembolism, fall into the highest risk category for recurrence [4–7].

D-dimer is a product of fibrin degradation by plasmin and is commonly used as a sensitive marker in clinical practice to rule out PE or DVT. As a degradation product, D-dimer reflects the extent of coagulation and fibrinolysis. It is associated with hypercoagulable states and can be easily followed through time, allowing it to be used as a prognostic tool; it also helps with the decision to continue or extend therapy, and to predict the risk of VTE recurrence [8,9]. On the other hand, ultrasound of the deep veins plays a crucial role in the diagnostic evaluation of DVT. After a first episode, repeated ultrasound assessments can provide valuable information on the recanalization status, as well as thrombus extension or progression. Evidence regarding the prognostic value of these tests and their role in predicting recurrence after discontinuation of anticoagulation is limited and remains controversial, largely due to inter-reader variability and reliance on qualitative D-dimer results [9–10].

The optimal duration of anticoagulant therapy after a first episode of unprovoked venous thromboembolism (VTE) remains uncertain. Although anticoagulants are highly effective for the prevention and treatment of VTE, they can affect quality of life, increase healthcare costs, and carry a clinically important risk of bleeding. Multiple approaches have been evaluated to support individualized risk stratification, including biomarkers, clinical prediction rules, and compression ultrasonography (CUS). To date, findings have been contradictory and inconclusive.

It also remains unclear whether these prognostic factors are predictive. Within the PROGRESS framework a predictive factor is a prognostic factor that modifies the effect of a treatment on an outcome [11]. In this context, different D-dimer levels (e.g., higher vs lower) may be associated with different effect sizes of extended anticoagulation after completion of standard-duration therapy.

Therefore, this comprehensive review was undertaken to identify the best available evidence regarding the utility of D-dimer and compression ultrasonography (CUS) as prognostic factors for venous thromboembolism (VTE) recurrence. In addition, we specifically evaluated the role of D-dimer as a predictive factor.

## Materials and methods

This systematic review was developed according to the recommendations of the Cochrane Collaboration and the PRISMA statement. Since this work concerns a prognosis factor, the PROGRESS framework was also followed.

The pre-specified protocol of this publication was registered and published in PROSPERO in October 2020 (ID: CRD42022341082, available at: https://www.crd.york.ac.uk/prospero/export_details_pdf.php). The PROGRESS framework was also followed.

### Search strategy and data sources

A systematic literature search was performed in MEDLINE, EMBASE, and The Cochrane Library through 28 May 2022, and the search was subsequently updated on 16 April 2025. The search strategy applied in each database used a combination of subheading terms and free words related to PET, DVT, D-dimer, and CUS. The detailed search is presented in S1 Table. The search was not limited by language, date of publication, or geographic location.

### Study selection

Two reviewers (S.S.M. and A.J.B.C.) independently screened titles and abstracts manually using the free version of the Ryann tool. No AI-powered tools were employed at any stage of the selection or extraction process. Discrepancies were resolved through discussion or third-party evaluation by S.M.R.

We included randomized and nonrandomized studies involving adults (≥18 years), regardless of sex or race/ethnicity, from any country, with an objectively confirmed diagnosis of a first episode of unprovoked deep vein thrombosis (DVT) or pulmonary embolism (PE) based on imaging confirmation (e.g., Doppler ultrasonography, angiography, or computed tomography, as appropriate). Unprovoked VTE was defined as occurring in the absence of major transient provoking risk factors. Studies were required to include objective confirmation of recurrent DVT or PE during follow-up using imaging methods to determine outcomes, and to report at least one D-dimer measurement or lower-limb ultrasonography performed at baseline, during anticoagulation, or after anticoagulation therapy as a prognostic factor.

D-dimer levels were analyzed as either continuous or dichotomous variables, adhering to the thresholds established in the original studies. In cases where multiple cut-off points were reported, the lowest level was used as the reference category, while the remaining test groups were collapsed into a single exposure category. Residual vein thrombosis via lower-limb ultrasonography was treated as a dichotomous variable (recanalization present versus absent), consistent with the criteria defined by the study authors

We also included studies that evaluated the predictive capacity of D-dimer. We considered two study designs: (1) randomized controlled trials assessing the effect of extended anticoagulation that reported effect estimates stratified by D-dimer levels or examined D-dimer as an interaction (effect-modification) factor; and (2) observational studies that included an interaction term between D-dimer level (high vs low or continuous concentration) and treatment duration (extended vs standard-duration anticoagulation) in multivariable regression models. Eligibility criteria for the population, D-dimer measurement, and outcome ascertainment were consistent with those described above.

Key exclusion criteria included studies enrolling patients with cancer, thrombosis at unusual sites, patients who did not complete at least three months of anticoagulation, or those with an indication for indefinite therapy.

If more than one study reported information from the same population, both were included if the intervention or follow-up duration was different. Discrepancies in study inclusion were resolved through discussion.

### Extraction

The search results were managed using Ryann in its free version. Records for the same studies were removed, and relevant studies were reviewed as full texts. Two authors (S.S.M and A.J.B.C) extracted information from eligible studies. Discrepancies in extracted data were resolved through discussion. The information collected included study characteristics, methodology, setting, participant descriptions (DVT, PE, major bleeding, anticoagulation duration, D-dimer or US); details of the intervention and comparison (D-dimer and/or US, type of measurement used, definition of positive/negative CUS); and results.

### Risk of bias in individual studies

Risk of bias of each individual study was assessed using the QUIPS (Quality in Prognosis Studies) tool [12], which consists of six domains: source of information analysis, attrition, participant selection, confounders, prognostic strategy, and data reporting. Judgment was made by consensus of two reviewers (S.S.M and A.J.B.C).

### Information synthesis

For dichotomous data, results are presented as a summary odds ratio (OR) or hazard ratio (HR) with 95% confidence intervals (CI). Statistical heterogeneity was assessed using I^2^ and χ^2^ test values and predictive intervals; substantial heterogeneity was considered to exist when I2 was greater than 40% and when a low p-value of less than 0.10 was found in the χ^2^ test. A random-effects meta-analysis with a generic inverse variance method was used to produce an overall summary because, clinically, some degree of heterogeneity between studies was expected.

The meta-analysis represents the pooled effect of raw data when adjusted effect estimates were unavailable. At the same time, associated measures were synthesized in a narrative way.

For meta-analysis of more than 10 studies, the plan was to conduct a funnel graph asymmetry assessment and to implement a formal test to explore publication bias. Otherwise, an exploratory analysis was performed through visual inspection. Subgroup analyses were planned according to the time of measurement of the index test. GRADEpro (GRADE Working Group, McMaster University, ON, Canada) was used to prepare the “Summary of Findings Tables.” The GRADE methodology assesses the overall quality of the body of evidence for each outcome according to risk of bias, consistency of effect, imprecision, directivity, and publication bias criteria. This methodology was adjusted for a prognosis study according to the guidelines reported by

## Results

**Fig 1:**
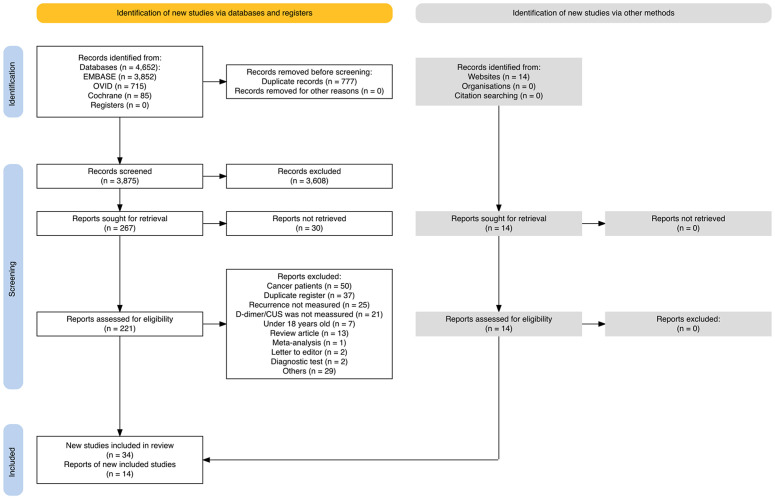
PRISMA flow diagram. Identification and screening of studies via databases and other methods.

The database search yielded 4,652 articles, while an additional 14 were identified through web searches and snowballing. After removing duplicates, 3,875 records were screened. Full-text reports were sought for retrieval, leaving 235 articles (221 from databases and 14 from websites) to be assessed for eligibility, as 30 reports could not be retrieved. Following the full-text assessment, 187 reports were excluded, resulting in 48 studies that met the inclusion criteria for the review. Of these, 27 studies were included in the meta-analysis, and six were analyzed using hazard ratios (HR) and time-to-event methods (Fig 1). Details of the included and excluded studies are provided in S2 Table.

### Study characteristics

The characteristics of the included studies are shown in Table 1. From the selected studies, 31 prospective cohorts [15,18–20,23–25,27–32,34,36–39,41–43,47–49,52,54,55,57–59], 10 clinical trials [16,17,21,22,26,35,40,44,45], 6 retrospective cohorts [13,33,46,50,53,56] and 1 case-control study [51] were obtained, for a total of 22252 patients over 18 years of age with a first PE or DVT event. The studies selected patients from China [13,53], UK [15,16,26–28,51], Australia [17], Austria [23,24,46,55], Italy [18–21,35–37,39–42,45,58], France [22], Spain [25], Canada [26–29,31,43,44], USA [26–29,44], Thailand [30], Brazil [32,33], Poland [34,54], Argentina [38], Iran [47], The Netherlands [48,49,51,59], Switzerland [44,50], and Russia [52]. We included nine studies that looked at D-dimer and US [18,19,25,27,32,33,37,38,42], 31 D-dimer [13–17,20,22–24,28–31,34–36,43,44,46–57,59], and eight studies which looked only at US [15,21,26,39–41,45,58]; twenty-seven studies with DVT or PE [14–16,18,20,21,23–30,34–37,42–44,46,49–52,55–57], 13 with DVT [17,19,31–33,38–41,45,47,48,58], and 4 exclusively with PE [13,22,53,54}(Table 1).

**Table 1:** Baseline characteristics.

Article identifier	Study type	Country	Period		Size	% Women	Age	D-dimer/US	PE/DVT	Duration of anticoagulation	Outcome Measurement	
An,2020 [13]	Retrospective Cohort	China	2013		150	61%	66	D-Dimer	PE	3-6 months	Immunoturbidimetry	Continuous variable (normal 0–550 μg/L)
Avnery,2019 [14]	Retrospective cohort	International Registration	2001-2008		460	59%	55	D-Dimer	PE/DVT	6-12 months	Immunoturbidimetry	Dichotomized (lab cut)
Baglin,2008 [15]	prospective cohort	UK	2001-2003		272	48%	65	US	PE/DVT	6-12 months	Latex	Dichotomized (500 ng/mL cut)
Bradbury,2019 [16]	clinical trial	UK	2011-2015		281	67.4%	62	D-Dimer	PE/DVT	3-6 months	Immunochromatography	Dichotomized (Cut ≥ 0.5 μg/ml)
Brighton,2017 [17]	clinical trial	Australia, New Zealand	2003-2011		822	45%	54	D-Dimer	DVT	6-24 months	CUS	RVT absent or present, does not define criteria
Cosmi,2005 [18]	prospective cohort	Italy	1995-2004		479	–	–	D-dimer and US dimer	PE/DVT		ELISA	Dichotomized (above 500 ng/ml) RVT according to Prandoni et al.
Cosmi,2008 [19]	prospective cohort	Italy	1995-2004		336	40%	71	D-dimer and US dimer	DVT	6-12 months	ELISA	Dichotomized (above 500 ng/ml) RVT according to Prandoni et al.
Cosmi,2010 [20]	prospective cohort	Italy	2005-2008		335	45.6%	62	D-Dimer	PE/DVT	6-12 months	Immunochromatography	Normal vs Abnormal (Qualitative)
Cosmi,2010b [21]	clinical trial	Italy	2002-2005		619	66%	66	US	PE/DVT	3 months	CUS	RVT definition according to Prandoni et al.
Couturaud, 2015 [22]	clinical trial	France	2007-2012		374	50%	58	D-Dimer	PE	6-18 months	ELISA/Immunoturbidimetry	Continuous variable
*Eichenger,2003* [23]	*prospective cohort*	Austria	1999-2002		175	–	–	D-Dimer	PE/DVT		ELISA	Quartiles (<250,250–500,500–750, > 750)
Eichinger,2010 [24]	prospective Cohort	Austria	1992-2008		929	40%	54	D-Dimer	PE/DVT	6-12 months	ELISA	Dichotomized (Cut-off ≥0.5 mg/L)
Franco-Moreno 2016 [25]	prospective cohort	Spain	2004-2013		398	45%	61	D-dimer and US dimer	PE/DVT	6-12 months	Not specified	Normal vs Abnormal (Qualitative)
Kearon, 1999 [26]	clinical Trial	Canada, UK, US	1994-1997		204	40%	59	US	PE/DVT	3-6 months	CUS	RVT definition according to Prandoni et al.
Kearon, 2015 [27]	Prospective Cohort	Canada, USA, UK, Ireland	2008-2012		410	43%	51	D-dimer and US dimer	PE/DVT	3-months	Immunochromatography	Normal vs Abnormal (Qualitative)
Kearon, 2016 [28]	prospective cohort	Canada, USA, UK,	–		285	43%	50	D-Dimer	PE/DVT	3-7 months	Immunochromatography	Normal vs Abnormal (Qualitative)
Kearon, 2019 [29]	prospeciva Cohort	Canada, USA	2008-2012		293	43%	51	D-Dimer	PE/DVT	3-6 months	Immunochromatography	Normal vs Abnormal (Qualitative)
Kijrattanakul, 2015 [30]	prospective cohort	Thailand	2013-2014		42	60%	53	D-Dimer	PE/DVT	3-6 months	ELISA	Dichotomized (D-dimer ≥ 500 ng/mL)
Legal,2011 [31]	prospective Cohort	Canada	2001-2006		452	49%	53%	D-Dimer	DVT	6-12 months	CUS	Normal – abnormal – inadequate (not specified)
Mazetto,2014 [32]	prospective cohort	Brazil	Not Described		52	–	–	D-dimer and US dimer	DVT	–	Immunoturbidimetry	Continuous variable
Mazetto,2018 [33]	cohorte retrospective	Brazil	2009-2011		56	62%	45	D-dimer and US dimer	DVT	3 months	Immunoturbidimetry	Dichotomized (630ng/mL Cut)
Mrozinska,2018 [34]	prospective Cohort	Poland	2008-2010		368	100%	44	D-Dimer	PE/DVT	>12 months	Not specified	Dichotomized (Cut > 250 ng/ml)
Palareti, 2006 [35]	clinical trial	Italy	2002-2005		627	66%	66	D-Dimer	PE/DVT	3 months	Immunochromatography	Normal vs Abnormal (Qualitative)
Palareti,2014 [36]	prospective cohort	Italy	2008-2011		1057	44%	66	D-Dimer	PE/DVT	3-6 months	ELISA	Cutting (Age, Sex, Measurement Technique)
Poli2008 [37]	prospective cohort	Italy	1996-2006		280	46%	62	D-dimer and US dimer	PE/DVT	3-6 months	Immunoturbidimetry	Dichotomized (<250 mcg/L)/ RVO transverse diameter >2mm or thrombus >40% of compressible area
Posadas-Martínez 2021 [38]	prospective cohort	Argentina	2015-2019		303	73%	80	D-dimer and US dimer	DVT	6 −12 months	CUS	Not Described
Prandoni, 2002 [39]	prospective cohort	Italy	1993-1996		313	44.4%	59	US	DVT	3 months	CUS	RVT according to Prandoni et al.
Prandoni2009 [40]	clinical trial	Italy	1999-2006		538	–	–	US	DVT	6-12 months	CUS	RVT according to Prandoni et al.
Prandoni,2015 [41]	prospective cohort	Italy	2003-2009		869	48%	63	US	DVT	3-6 months	CUS	RVT according to Prandoni et al.
Prandoni,2017 [42]	prospective cohort	Italy	2010−201		620	44%	61	D-dimer and US dimer	PE/DVT	3-6 months	Not specified	Dichotomized (250 and 500 μg/ml) RVT according to Prandoni et al..
Rodger,2008 [43]	prospective cohort	Canada	2001-2006		665	49%	53	D-Dimer	PE/DVT	3-6 months	ELISA	Continuous variable
Shrivastava,2006 [44]	clinical trial	USA, Canada, Switzerland	1999-2002		508	47%	56	D-Dimer	PE/DVT	3-6 months	Immunoturbidimetry	Dichotomized (>500 ng/mL)
Siragusa,2008 [45]	clinical trial	Italy	1999-2005		258	47%	57	US	DVT	3-6 months	CUS	DVT in case of persistent thrombus in two consecutive examinations.
Steinbrecher,2021 [46]	retrospective cohort	Austria	1998-2008		556	57%	47	D-Dimer	PE/DVT	3 months	Immunoturbidimetry	Continuous variable, D-dimer were log-base-2-transformed due to their skewed distribution.
Tamizifar, 2014 [47]	prospective cohort	Iran	2011-2012		68	50%	50	D-Dimer	DVT	6-12 months	ELISA	Dichotomized (500 ng/ml)
TenCatehoek,2011 [48]	prospective cohort	Netherlands	–		333	53%		D-Dimer	DVT	3-6 months	Not specified	Dichotomized (500 ng/ml)
Timp,2019 [49]	prospective cohort	Netherlands	1999-2004		4956	55%	48	D-Dimer	PE/DVT	3-6 months	Not specified	Continuous variable, natural logarithm D-dimer
Tritschler,2017 [50]	retrospective cohort	Switzerland	2009-2013		157	41%	74	D-Dimer	PE/DVT	6-12 months	ELISA	Dichotomized (500 ng/ml)
vanhylckamavlieg2015 [51]	cases & controls	UK & Netherlands	2003-2008		626	48%	53	D-Dimer	PE/DVT	3-6 months	ELISA/Latex	Dichotomized (500 ng/mL and 230 ng/mL)
Vorobyeva,2019 [52]	prospective cohort	Russia		–	112	68%	54	D-Dimer	PE/DVT		Not specified	Dichotomized (0.5 mcg/mL)
Yang,2020 [53]	retrospective cohort	China	2007-2018		597	46%	61	D-Dimer	PE	3-6 months	Not specified	Continuous variable, Log D-dimer (cut-off value = 3.436)
Zabczyk,2016 [54]	prospective cohort	Poland	2009-2012		156	47%	44	D-Dimer	PE	6-12 months	ELISA	Continuous variable,
Eichinger,2008 [55]	prospective cohort	Austria	1992-2005		861	44%	47	D-Dimer	PE/DVT	6-12 months	Immunoturbidimetry	Continuous variable D-dimer (per doubling)
Avnery,2020 [56]	Retrospective cohort	–	2018		1655	59%	55	D-dimer	PE/DVT	3-6 months	Not specified	Dichotomized (normal vs anormal)
Mronzinska, 2018b [57]	Prospective cohort	Poland	2008-2010		80	100%	44	D-dimer	PE/DVT	12 months	–	Continuos variable
Donmez2022 [58]	prospective cohort	Turkey			67			D’dimer	DVT	3-6 months		Dichotomized (age/100)
Iding2022 [59]	prospective cohort	Netherlands	2003-2018		825	49%	60	US	DVT	6-24 months		more than 2 mm in transversal diameter

CUS: compressive ultrasound, ELISA: enzyme-linked immunoassay, GSM: Grayscale median analyses, RVT: Residual Vein thrombosis. D-dimer measurement techniques: ELISA: Vidas® D-Dimer (Biomeriux), D-Dimer exclusion IITM (DEX2) (BIOMÉRIEUX); (Asserachrom D-dimer, Boehringer Mannheim, Germany); Immunoturmidimetry: IL Test D-Dimer (Instrumental Laboratory Spa), STA-Liatest-DD (Diagnostica Stago), Innovance D-dimer (Dade Behring); (BCS-XP, Siemens Healthcare, Marburg, Germany); Immunochromatography: Clearview Simplify D-dimer, Cobas H 232 D-dimer (Roche Diagnostic); Latex: MDAD D Dimer assay. HemosIL D-dimer HS; RVT measurement techniques: Prandoni et al (RVT when the diameter of the vein in the transverse plane on CUS is greater than > 4 mm), PE: Pulmonary Embolism

Different techniques were used for D-dimer measurement: 9 studies used assays based on immunoturbidimetry [13,14,22,32,33,37,44,46,55], 12 used ELISA [18,19,22–24,30,36,43,47,50,51,54], 2 used Latex [15,51] and 6 used immunochromatography [16,20,27–29,35]; other studies did not specify the measurement technique [25,34, 42,48,49,52,53,56]. In two studies, D-dimer was evaluated using different methods: ELISA and immunoturbidimetry [22] and ELISA and latex [51] (Table 1).

There were differences in the way outcomes were analyzed: 10 studies used D-dimer as a continuous variable [13,22,32,43,46,49,53–55,57], 14 studies dichotomized the result according to the laboratory cut-off or according to the age- and sex-adjusted cut-off point [14–16,18,19,24,30,33,34,37,42,44,47,48,50–52,56,58]. Six Studies used qualitative tests with positive or negative reports [20,25,27–29,35]

D-dimer measurement was performed at different time points in the disease and treatment; after completion of anticoagulation therapy in a period up to 6 months, [13–15,19,23,24,30,33,34,37,44,46,48–51,54] during therapy [16,22,25,27,28,43,52] or on the day of completion of treatment [18,20,47,55]. In three studies, D-dimer measurement led to a change in anticoagulant therapy [29,35,42].

Ultrasound studies used CUS to define the presence of Residual Venous Thrombosis (RVT) using different definitions of RVT; some categorized it as absent or present [17,45], others used the definition of RVT proposed by Prandoni’s group (RVT when the diameter of the vein in the LUS in the transverse plane is greater than > 4 mm) [18,19,21,26,39–42]; others used their own definition [37]; one study used GSM (Grayscale median analyses) [32]; and two did not specify the form of measurement [25,38].

### Risk of bias

**Fig 2:**
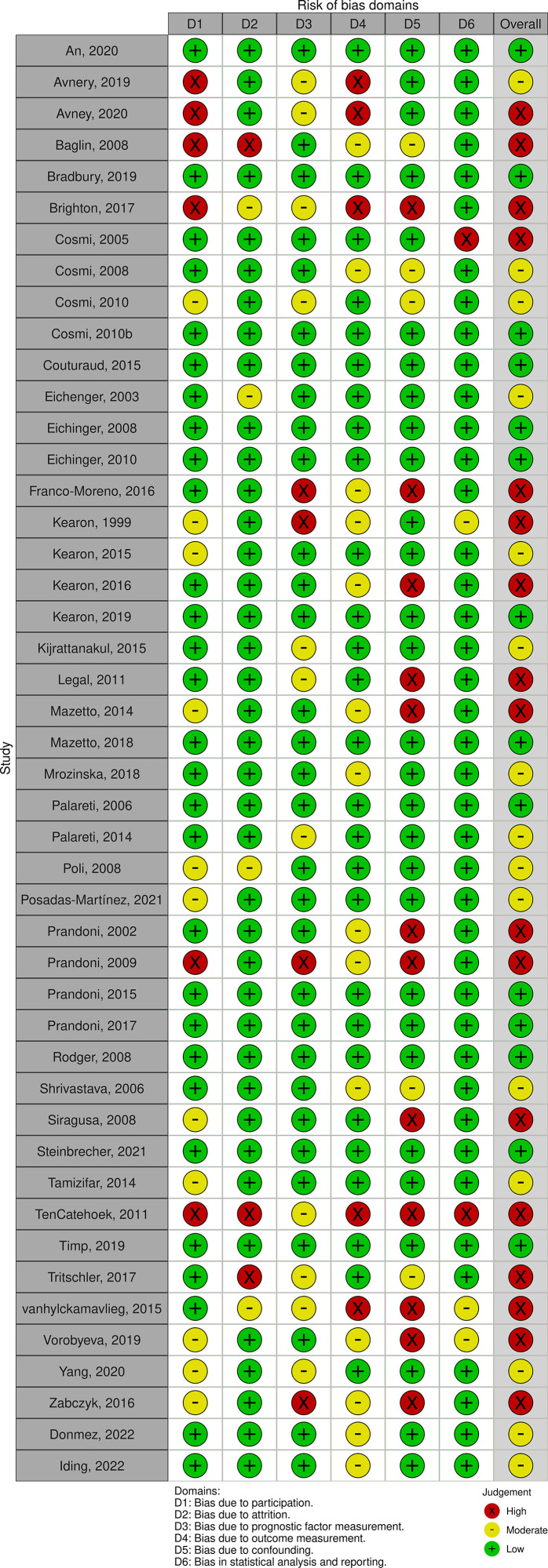
Risk of bias assessment across six domains (D1–D6) for the included studies. Green (+) indicates low risk of bias, yellow (−) indicates unclear risk, and red (×) indicates high risk of bias.

**Fig 3:**
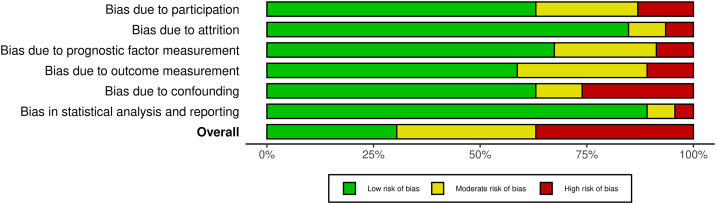
Risk of bias assessment across domains. Distribution of risk of bias across six domains: participation, attrition, prognostic factor measurement, outcome measurement, confounding, and statistical analysis/reporting.

Most studies were categorized as having moderate or high risk of bias (Figs 2 and 3). Regarding the overall judgment, seventeen studies were classified as high risk [15,17,18,25,26,28,31,32,39,40,45,48,50–52,54,56], while 15 were considered to have moderate risk or some concerns [14,20,23,27,30,34,36–38,44,47,53,57,59]. Finally, 16 studies were categorized as low risk of bias [13,16,19,21,22,24,29,33,35,41–43,46,49,55,58]. Regarding specific domains of bias, six studies were categorized as high risk for participation [14,15,17,40,48,56], while twelve showed moderate risk [20,26,27,32,37,38,45,47,52–54,59]. For attrition bias, three studies were classified as high risk [15,48,50] and four as moderate risk [17,23,37,51]. Bias in prognostic factor measurement was identified as high risk in four studies [25,26,40,54] and moderate in ten others [14,17,20,30,31,48,50,51,53,56]. Furthermore, outcome measurement bias was a concern in several reports, with five classified as high risk [14,17,48,51,56] and nine as moderate risk [15,25,32,34,50,52,54,58,59]. The most significant concerns were found in confounding factors, where twelve studies showed high risk [17,25,28,31,32,39,40,45,48,51,52,54] and seven were categorized as moderate risk [15,19,34,44,57–59]. Finally, bias related to statistical analysis and reporting was high in two studies [18,48] and moderate in twelve others [15,17,26,34,44,47,50–52,54,57,59] (Figs 2 and 3).

### Lower limb ultrasonography

**Fig 4:**
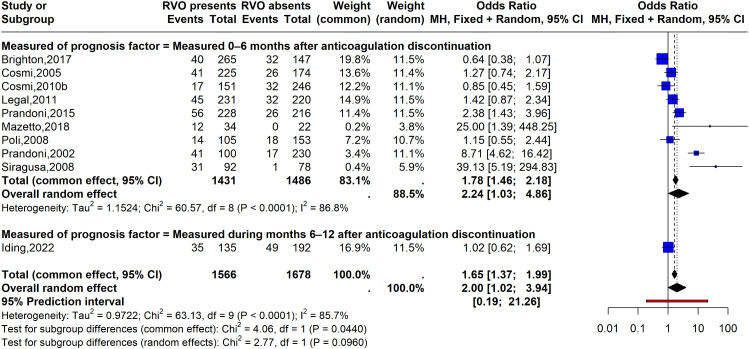
Forrest plot for RVT versus no RVT comparison. Outcome: Recurrent ETV. Subgroups represent the timing of measured of prognosis factor. The studies were categorized as 0-6 months after anticoagulation discontinuation and measured 6-12 months after coagulation discontinuation.

The main analysis was restricted to studies that assessed residual venous thrombosis (RVT) after completion of anticoagulant therapy [17,18,21,31,33,37,39,41,45,59]. Very low-certainty evidence suggested that residual thrombosis assessed by lower limb ultrasonography after a thrombotic event may be associated with an increased risk of recurrent VTE (OR 2.00, 95% CI 1.02 to 3.94, random-effects model; I² = 85.7%; 10 studies; prediction interval 0.21 to 18.14) (Fig 4). In the main analysis, we did not identify sources of heterogeneity. No subgroup differences were observed according to the timing of RVT measurement after anticoagulation discontinuation (test for subgroup differences: Chi² = 2.68, df = 1, P = 0.10104) (Fig 4), follow-up duration (test for subgroup differences: Chi² = 1.15, df = 1, P = 0.284), or sample size (test for subgroup differences: Chi² = 3.05, df = 1, P = 0.08). In an additional analysis, when studies that measured the prognostic factor during anticoagulation were included, RVT was also associated with recurrence (OR 1.82, 95% CI 1.01 to 3.01, random-effects model; I² = 83.1%; 13 studies; prediction interval 0.28 to 11.71). When retrospective cohort studies were excluded, the direction of the association remained the same, although the estimate was not statistically significant (OR 1.79, 95% CI 0.93 to 3.44, random-effects model; I² = 86.5%; 9 studies; prediction interval 0.18 to 17.83) (S1-S4 Figs).

### D-Dimer

**Fig 5:**
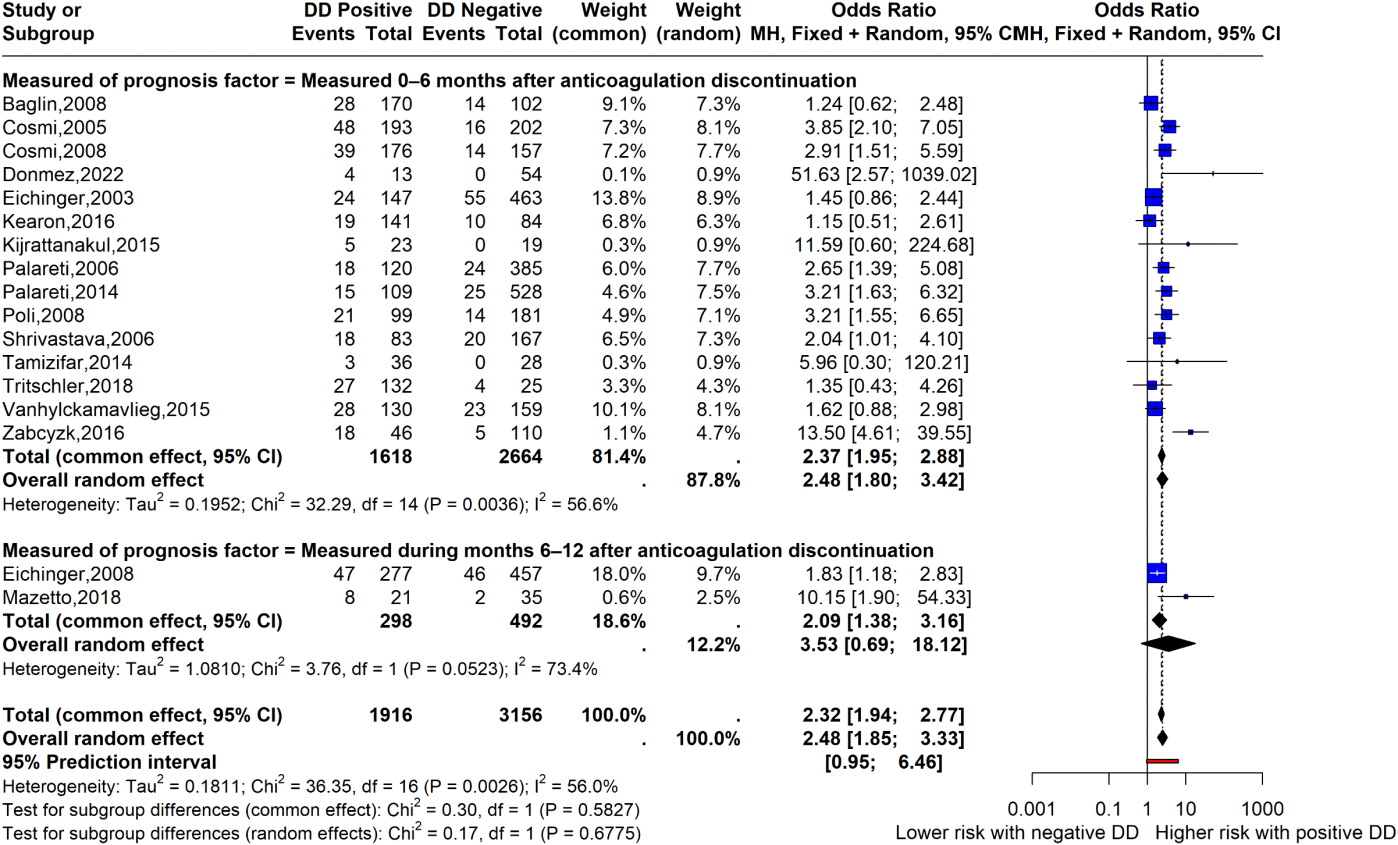
Forrest plot for normal vs abnormal D-dimer. D-dimer was measured after stopping anticoagulation. Outcome: Recurrent VTE. The studies were categorized as 0-6 months after anticoagulation discontinuation and measured 6-12 months after coagulation discontinuation.

The main analysis was restricted to studies in which D-dimer was assessed after anticoagulation discontinuation [15,18,19,23,28,30,32,33,35–37,44,47,50,51,54,55,58]. Very low-certainty evidence suggested that an abnormal D-dimer result after anticoagulation discontinuation following a thrombotic event was associated with an increased risk of recurrent VTE (OR, 2.48; 95% CI, 1.85–3.33; random-effects model; I² = 56.0%; prediction interval, 0.95–6.46; 17 studies). In the main analysis, the timing of D-dimer measurement (within the first 6 months after anticoagulation discontinuation vs 6–12 months after anticoagulation discontinuation) did not explain heterogeneity (test for subgroup differences under the random-effects model: χ² = 0.17, df = 1; P = 0.6775) (Fig 5). No significant subgroup differences were observed according to the D-dimer assay (test for subgroup differences: Chi² = 7.15, df = 5, P = 0.2) or follow-up duration (test for subgroup differences: Chi² = 0.05, df = 1, P = 0.83). However, a subgroup difference was observed according to sample size, comparing studies with fewer than 100 participants with those including more than 100 participants (test for subgroup differences: Chi² = 7.26, df = 1, P = 0.007) (S5–S7 Figs).

**Fig 6:**
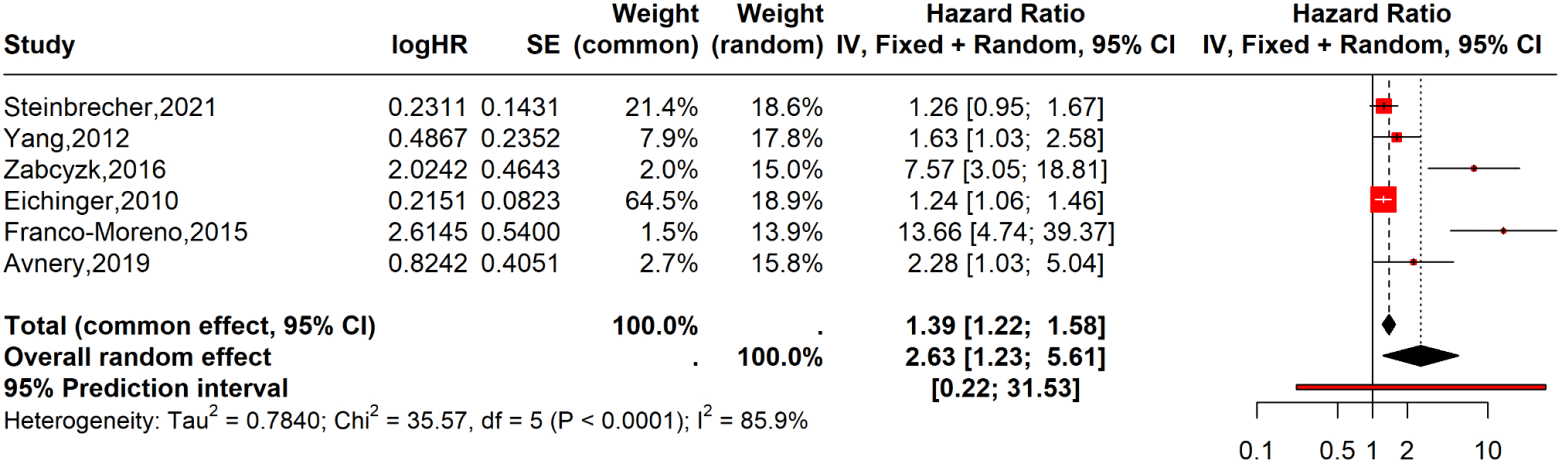
Forest plot normal vs abnormal D-dimer summarizing hazard ratios (HR). Summary of six studies evaluating time-to-event recurrence using a random-effects model.

Furthermore, a meta-analysis of six studies [14,24,25,46,53,54] evaluated the association between abnormal D-dimer levels and time to recurrence. Although the pooled analysis showed a significant increase in the hazard of recurrence (HR: 2.63; 95% CI: 1.23–5.61, I² = 86%; six studies), the evidence was graded as very low-certainty driven by substantial heterogeneity and wide prediction interval. (Fig 6).

### Predictive capacity of d-dimer

Two studies evaluated the predictive capacity of D-dimer. A randomized controlled trial of prolonged anticoagulation therapy found no statistically significant difference between the effect of prolonged anticoagulation between individuals with high versus low D-dimer levels (D-dimer ≥250ng/ml: RR 0.25 (95% CI, 0.07–0.89) vs D-dimer <250 ng/ml: RR 0.20 [0.06–0.71]; interaction p = 0.79). Other randomized that evaluated low intensity dose of warfarin or placebo found that patients with high or low levels of D-dimer had the same Hazard ratio of recurrence (D- dimer < 500 ng/ml: HR: 0.35 (95% CI, 0.15–0.83) vs D-dimer ≧ 500 ng/ml: HR 0.34 (95% CI, 0.14–0.82); interaction p = 0.95) [44]

### Sensitivity analysis

To assess the robustness of our findings regarding the prognostic value of D-dimer, we performed prespecified sensitivity analyses. First, the analyses were stratified by study design, with randomized controlled trials (RCTs) and prospective cohort studies evaluated separately. Second, we conducted a sensitivity analysis stratified by sample size (≥100 vs < 100 participants) to explore whether the magnitude of the association differed according to study size. No meaningful differences were observed in the association with D-dimer or in the consistency of the findings when analyses were restricted to prospective cohort studies or RCTs (OR, 2.38; 95% CI, 1.79–3.17; random-effects model; I² = 54.9%; prediction interval, 0.97–5.85; 16 studies) (S8 Fig). Likewise, when the analysis was restricted to studies with at least 100 participants, the effect estimate did not differ from that of the main analysis (OR, 2.26; 95% CI, 1.71–3.00; random-effects model; I² = 56.1%; prediction interval, 0.94–5.43; 13 studies) (S9 Fig). Given the effect modification introduced by including studies that measured D-dimer while participants were receiving anticoagulation, we also evaluated this scenario and found no differences in the effect estimate (OR, 2.39; 95% CI, 1.79–3.19; random-effects model; I² = 59.8%; prediction interval, 0.84–6.78; 20 studies) (S10 Fig).

### Publication bias

To assess publication bias, funnel plots were generated and are provided in the Supplementary Material (S11 Fig and S12 Fig). Since the interpretation of these plots relies on visual symmetry, we also performed Egger’s tests. In both cases, the p-value was greater than 0.05, indicating no significant evidence of publication bias.

### Certainty of the Evidence for D-Dimer and RV

The GRADE summary of findings for the prognostic value of D-dimer and RVT is provided in the Supplementary Material. Specifically, D-dimer is presented in S3 Table and RVT in S4 Table.

## Discussion

In this meta-analysis, an elevated D-dimer level after a thrombotic event was associated with a higher risk of recurrent VTE or PE. In addition, residual venous thrombosis (RVT) after a first VTE episode and anticoagulation therapy were associated with a higher risk of recurrent thromboembolic events.

There was substantial variability across studies in both the assay techniques used to measure D-dimer and the timing of measurement. By contrast, definitions of residual vein thrombosis (RVT) were broadly similar across studies. The duration of anticoagulation ranged from 3 to 12 months, according to different study-specific criteria. In subgroup analyses of the prognostic performance of D-dimer, neither the timing of measurement nor assay technique appeared to modify the effect. Similarly, for RVT, neither the timing of measurement nor follow-up duration explained the observed heterogeneity. However, for D-dimer, studies with fewer than 100 participants showed a different effect size than those with larger sample sizes. Nevertheless, the funnel plot and Egger test did not suggest publication bias, and no small-study effects were identified. For both D-dimer and RVT, the findings remained robust across sensitivity analyses by study design and after inclusion of studies in which prognostic factors were measured during anticoagulation. In addition, for D-dimer, restricting the analysis to studies with more than 100 participants did not change the main results.

Both meta-analyses demonstrated substantial between-study heterogeneity (D-dimer: I² = 56.0%; RVT: I² = 67.0%), which is an expected consequence of observational prognostic research, in which clinical and methodological variability across settings cannot be fully controlled. None of our prespecified subgroup analyses identified a significant source of heterogeneity, suggesting that the observed variability may reflect genuine between-population differences. To minimize this variability, our main analysis focused on populations in which participants were not receiving anticoagulation after measurement of the prognostic factor and included studies in which the prognostic factor was measured after anticoagulation discontinuation.

Interpreting heterogeneity solely based on I² thresholds is insufficient. As Borenstein (2023) has shown [60], I² is a ratio rather than an absolute measure of dispersion and does not convey the actual magnitude of effect variation across populations [60]. The prediction interval addresses this limitation more directly. For D-dimer, the prediction interval (0.95–6.46) indicates that, although the prognostic effect is expected to vary from a small, borderline association to a large increase in recurrence risk, an elevated post-anticoagulation D-dimer would still be expected to confer an increased risk of recurrence in most comparable populations, with the lower bound only marginally approaching the null. For RVT, the wider prediction interval (0.43–3.82) suggests an inconsistent prognostic effect, which may be partly explained by the operator-dependent nature of ultrasound-based thrombus detection.

When D-dimer and ultrasound (US) were evaluated as prognostic factors, their clinical utility remained uncertain. Using the GRADE approach, the certainty of evidence for D-dimer as a prognostic factor was very low. The certainty of evidence was downgraded primarily because of imprecision in the effect estimates and inconsistency across studies. The confidence interval around the point estimate for D-dimer and RVT ranged from a moderate to a large effect, making it difficult to determine the most plausible magnitude of the effect and, therefore, to advise patients about their risk of recurrence after an episode of VTE or PE. For this reason, we downgraded the certainty of evidence by one level. In addition, as noted above, the prediction interval showed substantial variability across studies for both prognostic factors. Most studies had at least one QUIPS domain rated as having moderate or high risk of bias. Taken together, these limitations resulted in very low certainty of evidence.

D-dimer has been proposed as a clinical tool to identify patients who might benefit from extended anticoagulation. However, the clinical trials retrieved in our review suggest that although patients with elevated D-dimer have a higher risk of thrombosis after discontinuing anticoagulation, D-dimer did not predict differential response to extended therapy. Across two trials, prolonged anticoagulation (at standard or reduced doses) produced similar effect estimates regardless of D-dimer status, and the treatment-by–D-dimer interaction for the outcome was not statistically significant. Therefore, consistent with the PROGRESS framework [11], D-dimer could appear to function as a prognostic factor rather than a predictive factor for extended anticoagulation.

Thromboembolic disease is a common condition with a high burden of morbidity and mortality. Moreover, it is a chronic entity with a high risk of recurrence, and although anticoagulation is an effective strategy, it entails a risk of complications and a risk of affecting quality of life. That is why, in the search for alternatives to help characterize individuals with a higher risk of recurrence, D-dimer and US, two tools commonly used in the diagnostic algorithm of VTE, have attracted the attention of researchers in search of their utility in the follow-up of patients and as prognostic determinants [4–6].

The different scores (DASH, HERDOO2) developed in an attempt at improving the characterization of patients and individualizing those who are candidates for extended anticoagulation therapy, appear to have limited usefulness in clinical practice despite extensive research [61–63].

To the best of our knowledge, this is the first study to analyze the available evidence regarding D-dimer and US as prognostic and predictive factors of recurrent VTE. Following a methodology different from a review of diagnostic tests, the data obtained are useful because they synthesize the evidence on the utility of these tools, known primarily for their role in the diagnostic algorithm, and now analyzed as predictive and forecasting strategies. The data presented suggest that, although D-dimer positivity and the presence of DVT were associated with an increased risk of recurrence, there are limitations to the efficacy of these tests as prognostic tools and their usefulness in defining changes in anticoagulation therapy.

Our study has several limitations. The first is that despite conducting an exhaustive search, it was not possible to review the full text of a significant number of studies initially selected by title and abstract, due to the date of publication and the fact that no response was received from the authors although we tried to reach out to them by e-mail. Second, heterogeneity between studies was significant. Finally, we must also consider the different measurement techniques, usefulness, cut-off points, different RVT definitions of RVT measurement timing, and the fact that the follow-up duration was not homogeneous between the studies.

Based on the results of this study, we suggest that D-dimer and RVT are associated with an increased risk of recurrence; however, their usefulness as prognostic factors is uncertain and therefore, the duration of anticoagulation should not be based on the outcome of these variables. The risk of recurrence will have to be balanced against the risk of bleeding if anticoagulation is extended

## Conclusions

The measurement of an abnormal D-dimer after stopping anticoagulation, as well as the presence of RVT at the time of treatment discontinuation, was associated with an increased risk of recurrence in the overall analysis. This study suggests that D-dimer and the presence of DVT are tools with limited prognostic capacity, therefore the decision to continue or discontinue anticoagulation should not be based on the result. Studies are needed to homogenize the time and type of measurement of these factors to improve their accuracy and their ability to better characterize the population at risk and help in decision-making regarding the duration of the anticoagulation therapy.

**Declaration of generative AI in scientific writing**: Grammarly v1.2.69.1350 was used during the preparation of this article to improve grammar and style. The authors reviewed and edited the content provided by these tools and take responsibility for it in the publication.

## Supporting information

S1 Table Search strategies applied in PubMed, Embase, Cochrane Library, and other databases. The table summarizes keywords, Boolean operators, and filters used for study identification.
(DOCX)

S2 Table Studies included and excluded in the systematic review. The table provides details on the reasons for exclusion and the final set of included studies.
(DOCX)

S3 Table GRADE evidence profile for the prognostic value of residual vein thrombosis (RVT) detected by compression ultrasonography (CUS) in predicting recurrent venous thromboembolism. (DOCX)

S4 Table GRADE evidence profile for the prognostic certainty of elevated D-dimer levels. (DOCX)

S1 Fig Forest plot of the subgroup analysis according to follow-up duration for residual venous thrombosis. (TIF)

S2 Fig Forest plot of the subgroup analysis according to sample size for residual venous thrombosis. (TIF)

S3 Fig Forest plot of the sensitivity analysis including studies in which residual venous thrombosis was measured during anticoagulation. (TIF)

S4 Fig Forest plot of the sensitivity analysis restricted to randomized controlled trials and prospective cohort studies for residual venous thrombosis. (TIF)

S5 Fig Forest plot of the subgroup analysis according to D-dimer assay type. (TIF)

S6 Fig Forest plot of the subgroup analysis according to follow-up duration for D-dimer. (TIF)

S7 Fig Forest plot of the subgroup analysis according to sample size (<100 vs ≥ 100 participants) for D-dimer. (TIF)

S8 Fig Forest plot of the sensitivity analysis restricted to randomized controlled trials and prospective cohort studies for D-dimer. (TIF)

S9 Fig Forest plot of the sensitivity analysis according to sample size (<100 vs ≥ 100 participants) for D-dimer. (TIF)

S10 Fig Forest plot of the sensitivity analysis including studies in which D-dimer was measured during anticoagulation. (TIF)

S11 Fig Funnel plot for publication bias assessment for residual venous thrombosis. (TIFF)

S12 Fig Funnel plot for publication bias assessment for D-dimer. (TIFF)
