# Supplementary material for: D-dimer and lower limb ultrasound as prognostic factors for recurrent deep venous thrombosis and pulmonary embolism: A systematic review and meta-analysis
Source: PLoS One. 2026 May 15;21(5):e0340158. doi: 10.1371/journal.pone.0340158 (PMC13178970; doi:10.1371/journal.pone.0340158)
Supplement: S1 Table — The table summarizes keywords, Boolean operators, and filters used for study identification. (DOCX) [file pone.0340158.s001.docx]

Ovid MEDLINE(R) ALL / PubMed(R) <1946 to Present>

1 exp Fibrin Fibrinogen Degradation Products/ 9876

2 (d adj2 dimer).ti,ab. 13827

3 (fibrin* adj3 degrada*).ti,ab. 4694

4 (fibrin* adj3 fragment*).ti,ab. 1217

5 1 or 2 or 3 or 4 20933

6 exp Ultrasonography/ 473181

7 Echotomograp*.ti,ab. 761

8 Echogra*.ti,ab. 10386

9 Sonography*.ti,ab. 33256

10 Ultraso*.ti,ab. 417358

11 doppler.ti,ab. 108660

12 6 or 7 or 8 or 9 or 10 or 11 725632

13 exp Venous Thrombosis/ 58196

14 exp Pulmonary Embolism/ 41906

15 (pulmonar* adj5 (embolism or t?romboembolism)).ti,ab. 41989

16 (veno* adj5 (t?rombo* or embolism)).ti,ab. 61104

17 14 or 15 59350

18 recurren*.ti,ab. 617911

19 Validat$.mp. or Predict$.ti. or Rule$.mp. or (Predict$ and (Outcome$ or Risk$ or Model$)).mp. or ((History or Variable$ or Criteria or Scor$ or Characteristic$ or Finding$ or Factor$) and (Predict$ or Model$ or Decision$ or Identif$ or Prognos$)).mp. or (Decision$.mp. and ((Model$ or Clinical$).mp. or Logistic Models/)) or (Prognostic and (History or Variable$ or Criteria or Scor$ or Characteristic$ or Finding$ or Factor$ or Model$)).mp. [mp=title, abstract, original title, name of substance word, subject heading word, floating sub-heading word, keyword heading word, organism supplementary concept word, protocol supplementary concept word, rare disease supplementary concept word, unique identifier, synonyms] 5586230

20 5 or 12 744662

21 13 or 14 or 15 or 16 138935

22 18 and 21 11234

23 20 and 22 1653

24 19 and 23 636

Embase

Session Results

.......................................................

No. Query Results Results Date

#47. #19 AND #46 3,300 28 May 2022

#46. #44 OR #45 14,898,936 28 May 2022

#45. (therap* OR treatment*) AND (guid* OR focus* OR 3,172,533 28 May 2022

direct* OR point* OR head*)

#44. #33 OR #43 13,209,646 28 May 2022

#43. #34 OR #35 OR #36 OR #37 OR #38 OR #39 OR #40 OR 1,346,665 28 May 2022

#41 OR #42

#42. 'multivariable':ti,ab 213,275 28 May 2022

#41. 'algorithm':ab,ti 289,854 28 May 2022

#40. 'indices':ab,ti 225,147 28 May 2022

#39. 'calibration':ab,ti 104,161 28 May 2022

#38. 'area under the curve' OR 'auc' 288,782 28 May 2022

#37. 'c statistic*' OR 'c-statistic*' 14,557 28 May 2022

#36. 'discriminate':ab,ti 88,693 28 May 2022

#35. 'roc curve'/exp 172,953 28 May 2022

#34. 'stratification':ab,ti 128,865 28 May 2022

#33. #25 OR #28 OR #29 OR #32 12,760,419 28 May 2022

#32. #24 AND #31 6,228,344 28 May 2022

#31. #25 OR #30 12,311,612 28 May 2022

#30. #26 OR #27 1,167,446 28 May 2022

#29. #20 OR #21 OR #22 3,570,266 28 May 2022

#28. prognostic AND (history OR variable* OR criteria 438,615 28 May 2022

OR scor* OR characteristic* OR finding* OR

factor* OR model*)

#27. 'logistic models'/exp 637,215 28 May 2022

#26. decision* AND (model* OR clinical*) 555,811 28 May 2022

#25. predict* OR model* OR decision* OR identif* OR 12,196,484 28 May 2022

prognos*

#24. history OR variable* OR criteria OR scor* OR 13,736,622 28 May 2022

characteristic* OR finding* OR factor*

#23. predict* AND (outcome* OR risk* OR model*) 1,643,423 28 May 2022

#22. rule* 241,985 28 May 2022

#21. predict* 2,619,882 28 May 2022

#20. validat* 1,008,601 28 May 2022

#19. #12 AND #18 5,598 28 May 2022

#18. #16 AND #17 27,175 28 May 2022

#17. recurren* 1,179,669 28 May 2022

#16. #13 OR #14 OR #15 233,176 28 May 2022

#15. veno* NEAR/5 (t?rombo* OR embolism) 112,775 28 May 2022

#14. pulmonar* NEAR/5 (embolism OR t?romboembolism) 70,634 28 May 2022

#13. 'venous thromboembolism'/exp 185,537 28 May 2022

#12. #5 OR #11 1,413,385 28 May 2022

#11. #6 OR #7 OR #8 OR #9 OR #10 1,377,781 28 May 2022

#10. 'doppler':ab,ti 161,943 28 May 2022

#9. ultraso* 784,861 28 May 2022

#8. echogra* 423,216 28 May 2022

#7. sonograph* 86,107 28 May 2022

#6. 'echography'/exp 953,848 28 May 2022

#5. #1 OR #2 OR #3 OR #4 44,319 28 May 2022

#4. fibrin* NEAR/3 fragment* 2,545 28 May 2022

#3. fibrin* NEAR/3 degradation* 9,073 28 May 2022

#2. 'd dimer':ab,ti 23,166 28 May 2022

#1. 'd dimer'/exp 33,287 28 May 2022

.......................................................

Update, 15 abril 2025

Database: Ovid MEDLINE(R) ALL <1946 to April 15, 2025>

Search Strategy:

--------------------------------------------------------------------------------

1 exp Fibrin Fibrinogen Degradation Products/ (10622)

2 (d adj2 dimer).ti,ab. (18529)

3 (fibrin* adj3 degrada*).ti,ab. (5039)

4 (fibrin* adj3 fragment*).ti,ab. (1240)

5 or/1-4 (25835)

6 exp Ultrasonography/ (508954)

7 echotomograp*.ti,ab. (764)

8 echogra*.ti,ab. (10888)

9 sonography*.ti,ab. (35285)

10 ultraso*.ti,ab. (500184)

11 doppler.ti,ab. (118429)

12 or/6-11 (825806)

13 5 or 12 (849411)

14 exp Venous Thrombosis/ (62125)

15 exp Pulmonary Embolism/ (45361)

16 (pulmonar* adj5 (embolism or t?romboembolism)).ti,ab. (49388)

17 (veno* adj5 (t?rombo* or embolism)).ti,ab. (72139)

18 or/14-17 (157738)

19 recurren*.ti,ab. (740349)

20 18 and 19 (12954)

21 13 and 20 (1833)

22 validat$.mp. (948592)

23 predict$.ti. (525316)

24 rule$.mp. (212700)

25 (Predict$ and (Outcome$ or Risk$ or Model$)).mp. (1524974)

26 ((History or Variable$ or Criteria or Scor$ or Characteristic$ or Finding$ or Factor$) and (Predict$ or Model$ or Decision$ or Identif$ or Prognos$)).mp. (5665541)

27 (Decision$ and (Model$ or Clinical$)).mp. (358387)

28 exp Logistic Models/ (156643)

29 (Prognostic and (History or Variable$ or Criteria or Scor$ or Characteristic$ or Finding$ or Factor$ or Model$)).mp. (353228)

30 or/22-29 (7036219)

31 21 and 30 (707)

32 limit 31 to yr="2022 -Current" (77)

Database: Embase Classic+Embase <1947 to 2025 April 15>

Search Strategy:

--------------------------------------------------------------------------------

1 exp D dimer/ (48094)

2 (d adj2 dimer).ti,ab. (31251)

3 (fibrin* adj3 degrada*).ti,ab. (6607)

4 (fibrin* adj3 fragment*).ti,ab. (1628)

5 or/1-4 (59203)

6 exp echography/ (1166559)

7 echotomograp*.ti,ab. (979)

8 echogra*.ti,ab. (15243)

9 sonography*.ti,ab. (49309)

10 ultraso*.ti,ab. (744793)

11 doppler.ti,ab. (179513)

12 or/6-11 (1581411)

13 5 or 12 (1628699)

14 exp venous thromboembolism/ (232897)

15 exp lung embolism/ (142735)

16 (pulmonar* adj5 (embolism or t?romboembolism)).ti,ab. (84419)

17 (veno* adj5 (t?rombo* or embolism)).ti,ab. (115870)

18 or/14-17 (287282)

19 recurren*.ti,ab. (1151917)

20 18 and 19 (27434)

21 13 and 20 (6011)

22 validat$.mp. (1348037)

23 predict$.ti. (733759)

24 rule$.mp. (299485)

25 (Predict$ and (Outcome$ or Risk$ or Model$)).mp. (2108354)

26 ((History or Variable$ or Criteria or Scor$ or Characteristic$ or Finding$ or Factor$) and (Predict$ or Model$ or Decision$ or Identif$ or Prognos$)).mp. (7807154)

27 (Decision$ and (Model$ or Clinical$)).mp. (699247)

28 exp statistical model/ (774327)

29 (Prognostic and (History or Variable$ or Criteria or Scor$ or Characteristic$ or Finding$ or Factor$ or Model$)).mp. (557624)

30 or/22-29 (9931869)

31 21 and 30 (2378)

32 limit 31 to yr="2022 -Current" (552)
