# Supplementary material for: D-dimer and lower limb ultrasound as prognostic factors for recurrent deep venous thrombosis and pulmonary embolism: A systematic review and meta-analysis
Source: PLoS One. 2026 May 15;21(5):e0340158. doi: 10.1371/journal.pone.0340158 (PMC13178970; doi:10.1371/journal.pone.0340158)
Supplement: S2 Table — The table provides details on the reasons for exclusion and the final set of included studies. (DOCX) [file pone.0340158.s002.docx]

| **Articles** | | |
| --- | --- | --- |
| **title** | **Included Yes/No** | **Reason for exclusion** |
| Is the predictive quality of D-dimer for the recurrence of thrombosis time dependent? | Yes |  |
| Recurrences after stopping anticoagulant therapy in Thai patients with non cancer-related venous thromboembolism | Yes |  |
| Residual vein thrombus does not predict for recurrent vein thrombosis in the aspire study | Yes |  |
| Elevated D-dimer level after 1 month anticoagulant therapy as a predictor for adverse outcomes in patients with venous thromboembolism: 10-year follow-up results | Yes |  |
| D-dimer, factor VIII coagulant activity, low-intensity warfarin and the risk of recurrent venous thromboembolism | Yes |  |
| Combination of D-dimer, F1+2 and residual vein obstruction as predictors of VTE recurrence in patients with first VTE episode after OAT withdrawal [4] | Yes |  |
| Annals of internal medicine, residual thrombosis on ultrasonography to guide the duration of anticoagulation in patients with deep venous thrombosis, A randomized trial | Yes |  |
| Lower echogenicity of the residual venous thrombosis at ultrasound examination is associated to recurrent thrombosis events | Yes |  |
| The impact of residual thrombosis on the long-term outcome of patients with deep venous thrombosis treated with conventional anticoagulation | Yes |  |
| Residual vein thrombosis and serial D-dimer for the long-term management of patients with deep venous thrombosis | Yes |  |
| Prediction of recurrent venous thrombosis in all patients with a first venous thrombotic event: The Leiden Thrombosis Recurrence Risk Prediction model (L-TRRiP) | Yes |  |
| D-dimer levels over time after anticoagulation and the association with recurrent venous thromboembolism | Yes |  |
| Long-term risk of recurrence in patients with a first unprovoked venous thromboembolism managed according to d-dimer results; A cohort study. | Yes |  |
| D-dimer levels and risk of recurrence following provoked venous thromboembolism: findings from the RIETE registry. | Yes |  |
| A randomised controlled trial of extended anticoagulation treatment versus standard treatment for the prevention of recurrent venous thromboembolism (VTE) and post-thrombotic syndrome in patients being treated for a first episode of unprovoked VTE (the ExACT study). | Yes |  |
| d-dimer is a predictor of clot resolution in patients with pulmonary thromboembolism: A retrospective cohort study. | Yes |  |
| Predictors of recurrence of venous thromboembolic disease after discontinuing of anticoagulation: a prospective cohort study. | Yes |  |
| Variable predictors of acute pulmonary embolism recurrence with duration of follow-up. | Yes |  |
| D-dimer levels and recurrence in patients with unprovoked VTE and a negative qualitative D-dimer test after treatment. | Yes |  |
| Usefulness of D-Dimer Testing in Predicting Recurrence in Elderly Patients with Unprovoked Venous Thromboembolism. | Yes |  |
| Prothrombotic Fibrin Clot Phenotype Is Associated With Recurrent Pulmonary Embolism After Discontinuation of Anticoagulant Therapy. | Yes |  |
| Residual Vein Thrombosis Echogenicity Is Associated to the Risk of DVT Recurrence: A Cohort Study. | Yes |  |
| Altered plasma clot properties increase the risk of recurrent deep vein thrombosis: a cohort study. | Yes |  |
| Unfavorably altered plasma clot properties in women with a HERDOO2 score equal to or greater than 2 and prediction of recurrent venous thromboembolism. | Yes |  |
| The prediction role of D-dimer in recurrence of venous thromboembolism 1-year after anticoagulation discontinuing following idiopathic deep vein thrombosis. | Yes |  |
| The risk of a first and a recurrent venous thrombosis associated with an elevated D-dimer level and an elevated thrombin potential: results of the THE-VTE study. | Yes |  |
| A risk score for prediction of recurrence in patients with unprovoked venous thromboembolism (DAMOVES). | Yes |  |
| Usefulness of repeated D-dimer testing after stopping anticoagulation for a first episode of unprovoked venous thromboembolism: the PROLONG II prospective study. | Yes |  |
| Residual venous obstruction, alone and in combination with D-dimer, as a risk factor for recurrence after anticoagulation withdrawal following a first idiopathic deep vein thrombosis in the prolong study. | Yes |  |
| Risk assessment of recurrence in patients with unprovoked deep vein thrombosis or pulmonary embolism: the Vienna prediction model. | Yes |  |
| Residual vein obstruction as a predictor for recurrent thromboembolic events after a first unprovoked episode: data from the REVERSE cohort study. | Yes |  |
| D-dimer levels in combination with residual venous obstruction and the risk of recurrence after anticoagulation withdrawal for a first idiopathic deep vein thrombosis. | Yes |  |
| **D-dimer testing to determine the duration of anticoagulant therapy.** | Yes |  |
| Unprovoked recurrent venous thrombosis: prediction by D-dimer and clinical risk factors. | Yes |  |
| D-dimer and factor VIII are independent risk factors for recurrence after anticoagulation withdrawal for a first idiopathic deep vein thrombosis. | Yes |  |
| Residual vein thrombosis to establish duration of anticoagulation after a first episode of deep vein thrombosis: the Duration of Anticoagulation based on Compression UltraSonography (DACUS) study. | Yes |  |
| Identifying unprovoked thromboembolism patients at low risk for recurrence who can discontinue anticoagulant therapy. | Yes |  |
| Prediction of recurrent venous thromboembolism by endogenous thrombin potential and D-dimer. | Yes |  |
| Residual venous thrombosis as a predictive factor of recurrent venous thromboembolism. | Yes |  |
| Monitoring of patients with deepvein thrombosis during and after anticoagulation with D-dimer | Yes |  |
| Yesx Months vs Extended Oral AnticoagulationAfter a First Episode of Pulmonary EmbolismThe PADIS-PE Randomized Clinical Trial | Yes |  |
| A Comparison of Three Months of Anticoagulation with Extended Anticoagulation for a First Episode of Idiopathic Venous Thromboembolism | Yes |  |
| D-dimer testing to select patients with a first unprovoked venous thromboembolism who can stop anticoagulant therapy: a cohort study | Yes |  |
| D-dimer to guide the duration of anticoagulation in patients with venous thromboembolism: a management study. | Yes |  |
| The role of D-dimer testing in the evaluation of DVT recurrence | Yes |  |
| Performance of D-Dimers in Patients with Prior History of Venous Thromboembolism Based on Anticoagulation Status | Yes |  |
| esidual Venous Obstruction as an Indicator of Clinical Outcomes following Deep Vein Thrombosis: A Management Study | Yes |  |
| Management of patients with venous thromboembolism and a high recurrence risk estimated by the Vienna Prediction Model: a prospective cohort study | Pending |  |
| A Neural Network Approach to Predict Recurrent VTE Based on Coagulation Parameters | Pending | An email was sent to the authors |
| DASH score for recurrent VTE-Retrospective study | Pending | An email was sent to the authors |
| Predictive value of D dimer in recurrent venous thromboembolism | Pending |  |
| Elevated D-dimer level predicts recurrent VTE | Pending |  |
| Post-treatment residual thrombus increases the risk of recurrent deep vein thrombosis? | Pending |  |
| Ultrasonography may help guide decisions to discontinue anticoagulation therapy for deep venous thrombosis | Pending |  |
| Residual thrombosis on ultrasonography to guide the duration of anticoagulation in patients with deep venous thrombosis. A randomized trial | Yes |  |
| D-dimer can predict risk of recurrent venous thromboembolism regardless of patient age, timing of testing, or characteristics of assay | Pending |  |
| Prospective study to identify risk factors for post thrombotic syndrome and evaluate role of compression ultrasound sonography to decide discontinuation of anticoagulant | Pending |  |
| Lower levels of circulating endothelial progenitor cells are associated with increased recurrence after unprovoked venous thrombosis | Pending |  |
| A trial to determine if withholding anticoagulation is not worse than standard anticoagulation therapy in the treatment of blood clots in the lungs | Pending |  |
| D-dimer Levels During and After Anticoagulation in Patients With a Previous Venous Thromboembolism: Effects on the Risk of Recurrence | Pending |  |
| Residual Vein Thrombosis and the Optimal Duration of Low Molecular Weight Heparin in Cancer Patients With Deep Vein Thrombosis | Pending |  |
| Residual Vein Thrombosis Establishes the Optimal Duration of Oral Anticoagulants | Pending |  |
| Extended anticoagulation for thrombosis patients showing an activated coagulation system as detected by a simple blood test | Pending |  |
| Clinical Decision Rule A Clinical Decision Rule to Identify Patients With "Unprovoked" Venous Thromboembolism Who Can Discontinue Anticoagulants After 6 Months of Treatment | Pending |  |
| D-dimer testing to assess the individual risk of venous thromboembolic recurrence in non-elderly patients of both genders: follow the rules!. | Pending |  |
| Risk factors for residual thrombotic occlusion after proximal deep vein thrombosis of the legs. | Pending |  |
| [Independent predictors of deep vein thrombosis (results of prospective 18 months study)]. | Pending |  |
| D-dimer levels < 250 ng/mL after oral anticoagulation predicted a low risk for recurrent venous thromboembolism. | Pending |  |
| The association of D-dimer levels with clinical outcomes in patients presenting with acute pulmonary embolism. | Pending |  |
| [High negative predictive value for recurrence of venous thromboembolism with d-dimer carried out three months after the suspension of treatment with oral anticoagulants in patients over 60]. | Pending |  |
| Risk of deep vein thrombosis recurrence: high negative predictive value of D-dimer performed during oral anticoagulation. | Pending |  |
| Potential use of D-dimer measurement in patients treated with oral anticoagulant for a venous thromboembolic episode. | Pending |  |
| Prospective 12-year follow-up study of clinical and hemodynamic sequelae after deep vein thrombosis in low-risk patients (Zurich study). | Pending |  |
| Extended treatment of venous thromboembolism | No | did not evaluate d-dimer as a prognostic factor |
| International Normalized Ratio Predicts Recurrence and Bleeding in Patients With Acute Venous Thromboembolism Who Undergo Direct Oral Anticoagulants | No | Cancer patients |
| Recurrent Venous Thromboembolism in Patients on Anticoagulation: An Update Based on the Revised AWMF S2k Guideline | No | Review |
| Aetiological, clinical and therapeutic prognostic factors for the evolution of deep vein thrombosis followed up with serial venous Doppler ultrasound | No | provocated events |
| Clinical profile, risk factors, and clinical outcomes in patients of venous thromboembolism at a tertiary care center | No | Cancer patients |
| Characteristics and Outcomes of Patients Consulted by a Multidisciplinary Pulmonary Embolism Response Team: 5-Year Experience | No | did not evaluate d-dimer as a prognostic factor |
| Risk for Recurrent Venous Thromboembolism in Patients With Subsegmental Pulmonary Embolism Managed Without Anticoagulation A Multicenter Prospective Cohort Study | No | did not evaluate d-dimer as a prognostic factor |
| Cost-effectiveness of performing reference ultrasonography in patients with deep vein thrombosis | No | did not evaluate d-dimer as a prognostic factor |
| Long-term anticoagulation decisions in men over 50 years old following a first unprovoked venous thrombosis is not aided by the DASH score | No | did not evaluate d-dimer as a prognostic factor |
| Effectiveness and Safety of Direct Oral Anticoagulants vs. Warfarin and Recurrence After Discontinuation in Patients With Acute Venous Thromboembolism in the Real World | No | Cancer patients |
| Long-Term Outcomes in Two-Year Follow-Up after Primary Treatment in Patients with a Prior Venous Thromboembolic Event: A Prospective, Observational, Real-Life Study | No | did not evaluate d-dimer as a prognostic factor |
| Different cut-off values of quantitative D-dimer (DD) assays to establish duration of oral anticoagulation treatment (OAT) after venous thromboembolism (VTE) | No | (duplicated PROLONG 2) |
| Association of D-Dimer test at hospital discharge with recurrent venous thromboembolism events in patients with acute pulmonary embolism | No | did not specified anticoagulation time |
| Risk of recurrent venous thrombosis associated with elevated D-dimer levels | No | (duplicated VTE trial) |
| Risk of recurrent venous thromboembolism in patients with a first episode of unprovoked pulmonary embolism: A pre-specified subgroup analysis from the padis pe randomized trial | No | (duplicated PADISPE) |
| D-dimer and comorbidities as risk factors for recurrence after a first episode of venous thromboembolism in the extended follow-up of the prolong study | No | (duplicated PROLONG 1) |
| The REVERSE I and II studies: Impact of using. Men continue and HERDOO2 clinical decision rule to guide anticoagulant therapy in patients with first unprovoked venous thromboembolism | No | (duplicated REVERSE) |
| High rate of unprovoked recurrent venous thrombosis is associated with high thrombin-generating potential in a prospective cohort study. | No | (Duplicated Cambdridge II) |
| Optimal duration of oral anticoagulant therapy after a first episode of venous thromboembolism: Where to go? | No | Review |
| Can anticoagulant treatment be tailored with biomarkers in patients with venous thromboembolism? | **No** | Review |
| Value of D-dimer testing to decide duration of anticoagulation after deep vein thrombosis: Not yet | No | Review |
| Value of D-dimer testing to decide duration of anticoagulation after deep vein thrombosis: Yes | No | Review |
| Thrombosis: Duration of anticoagulation after VTE: guided by ultrasound? | No | Review |
| Thrombosis: A new scoring system for simple risk prediction in patients with unprovoked venous thromboembolism | No | Review |
| Editorial comment: D-dimer to guide long-term anticoagulant treatment duration - Have we gotten anywhere? | No | Review |
| Prolonged anticoagulation in VTE with direct oral anticoagulants: Towards an individual analysis of net clinical benefit? | No | Review |
| Duration of anticoagulant treatment for unprovoked deep-vein thrombosis - is prolonged long enough? | No | Review |
| D-dimer testing after anticoagulant discontinuation to predict recurrent venous thromboembolism | No | Review |
| d-Dimer testing to predict recurrence risk in venous thromboembolism: looking for a useful threshold: a rebuttal. | No | Review |
| Predicting disease recurrence in patients with previous unprovoked venous thromboembolism: a proposed prediction score (DASH). | No | Review |
| D-dimer to predict thrombosis recurrence after unprovoked venous thromboembolism: Effect of patient-and D-dimer-related factors on recurrence prediction | No | metaanalisis |
| Utility of a recurrence prediction tool (DASH score) at a single centre after unprovoked venous thromboembolism: patient uptake of the tool and short term risks of stopping anticoagulation. | No | Letter to the Editor. |
| Identifying patients at risk of recurrent venous thromboembolism. | No | Letter to the Editor. |
| To describe D-Dimer levels in patients receiving direct oral anticoagulants with and without suspected recurrence of venous thromboembolism | No | Diagnostic d dimer |
| D-dimer is useful in the assessment of suspected recurrent venous thromboembolism in patients on rivaroxaban or apixaban | No | Diagnostic d dimer |
| Combined D-dimer and clinical probability are useful for exclusion of recurrent deep venous thrombosis. | No | Diagnostic d dimer |
| Risk prediction of recurrent venous thrombosis; where are we now and what can we add? | No | duplicated |
| Risk factor for the prediction of recurrent venous thromboembolism | No | duplicated |
| Influence of chronic heart failure on the risk of deep vein thrombosisrecurrence, hemorrhagic complications and D-dimer level during anticoagulant therapy in patients with venous thromboembolism | No | duplicated |
| Residual vein thrombosis as a strong predictor of recurrent thromboembolism and post-thrombotic syndrome: A prospective cohort study | No | duplicated |
| D-dimer to select patients with a first unprovoked venous thromboembolism (VTE) who have anticoagulants stopped at 3-7 months or have treatment continued indefinitely: A multicentre management study | No | duplicated |
| D-dimer testing during anticoagulant therapy should be used to indicate patients who need extended anticoagulant therapy | No | duplicated |
| Fibrin clot phenotype in patients with pulmonary embolism: Association with recurrent venous thromboembolism following discontinuation of anticoagulant therapy | No | duplicated |
| Predicting recurrence in elderly patients with unprovoked venous thromboembolism: Prospective validation of the updated Vienna Prediction Model | No | duplicated |
| D-dimer level after one month of initial anticoagulant therapy determines the efficacy of long-term warfarin therapy | No | duplicated |
| Increased D-Dimer levels and residual venous thrombosis are associated with late recurrence of deep venous thrombosis | No | duplicated |
| Predictors of recurrence of venous thromboembolic disease after suspension of anticoagulation | No | duplicated |
| Incidence of recurrent venous thromboembolism and of chronic thromboembolic pulmonary hypertension in patients after a first episode of pulmonary embolism. | No | duplicated |
| Residual thrombosis on ultrasonography to guide the duration of anticoagulation in patients with deep venous thrombosis: a randomized trial. | No | duplicated |
| The long-term recurrence risk of patients with unprovoked venous thromboembolism: An observational cohort study | No | duplicated |
| Impact of Asymptomatic Pulmonary Embolism on the Long-Term Prognosis of Patients with Deep Venous Thrombosis. | No | duplicated |
| Retrospective review of D-dimer testing for venous thrombosis recurrence risk stratification: is this a useful test in the real world?. | No | under 18 |
| The risk of ipsilateral versus contralateral recurrent deep vein thrombosis in the leg | No | under 18 |
| Post-thrombotic syndrome, recurrence, and death 10 years after the first episode of venous thromboembolism treated with warfarin for 6 weeks or 6 months | No | under 18 |
| The Duration of Oral Anticoagulant Therapy after a Second Episode of Venous Thromboembolism | No | under 18 |
| A Comparison of Yesx Weeks with Yesx Months of Oral Anticoagulant Therapy after a First Episode of Venous Thromboembolism | No | under 18 |
| A prospective study of quantitative D-dimer and endogenous thrombin potential measurements in individuals stopping oral anticoagulants for acute venous thromboembolism; role in predicting risk of recurrent thrombotic events | No | under 18 |
| Incidence of recurrent venous thromboembolism and of chronic thromboembolic pulmonary hypertension in patients after a first episode of pulmonary embolism | No | under 18 |
| Predicting the risk of recurrent venous thromboembolism in patients with cancer: A prospective cohort study. | No | Cancer patients |
| A high Gas6 level in plasma predicts venousthromboembolism recurrence, major bleeding and mortality inthe elderly: a prospective multicenter co | No | Cancer patients |
| Risk of recurrence in patients with pulmonary embolism: Predictive role of d-dimer and of residual perfusion defects on lung scintigraphy | No | Cancer patients |
| Prognostic value of D-dimer in patients with venous thromboembolism | No | Cancer patients |
| Tissue factor (TF) as predictor of recurrent venous thromboembolism (VTE): risk factor and biomarker analysis from the catch trial of treatment of cancer-associated VTE with tinzaparin or warfarin | No | Cancer patients |
| Brain natriuretic peptide, troponin and D-dimer levels in relation to long-term functional outcome after a first episode of pulmonary embolism: Results from the E.L.O.P.E. study | No | Cancer patients |
| Outpatient treatment for PE: Risk factors for mortality, recurrent VTE and pulmonary hypertension at 6 month | No | Cancer patients |
| Dynamics of case-fatalilty rates of recurrent thromboembolism and major bleeding in patients treated for venous thromboembolism | No | Cancer patients |
| Erratum: Risk of venous thromboembolism recurrence: High negative predictive value of D-dimer performed after oral. Anticoagulation is stopped (Thromb Haemost (2002) 87 (7-12)) | No | Cancer patients |
| The long term clinical course of acute deep vein thrombosis of the arm: Prospective cohort study | No | Cancer patients |
| Acute reactives and markers of inflammation in venous thromboembolic disease: Clinical and evolution outcomes | No | Cancer patients |
| High D-dimer levels at presentation in patients with venous thromboembolism is a marker of adverse clinical outcomes | No | Cancer patients |
| Does D-dimer predict recurrence of venous thromboembolism in elderly patients? | No | Cancer patients |
| Clinical profile of patients with proximal deep venous thrombosis patients-a prospective observational study from a tertiary care hospital in North India | No | Cancer patients |
| Recurrent deep vein thrombosis after the first venous thromboembolism event: A single-institution experience | No | Cancer patients |
| Pulmonary embolism: Association between deep vein thrombosis, clinical profile and long-term outcome | No | Cancer patients |
| Deep Venous Thrombosis Recurrence and Its Predictors at Selected Tertiary Hospitals in Ethiopia: A Prospective Cohort Study | No | Cancer patients |
| Long-term outcomes in patients with pulmonary embolism: results from a longitudinal cohort study | No | Cancer patients |
| A significant decrease in D-dimer concentration within one month of anticoagulation therapy as a predictor of both complete recanalization and risk of recurrence after initial pulmonary embolism | No | Cancer patients |
| Joint analysis of D-dimer, N-terminal pro b-type natriuretic peptide, and cardiac troponin I on predicting acute pulmonary embolism relapse and mortality | No | Cancer patients |
| Elevated plasma D-dimer levels are associated with risk of future incident venous thromboembolism | No | Cancer patients |
| Ethiological, Clinical and Therapeutic Prognostic Factors for the Evolution of Deep Vein Thrombosis Followed-Up by Serial Venous Doppler Ultrasound | No | Cancer patients |
| Principal Component Analysis on Recurrent Venous Thromboembolism. | No | Cancer patients |
| Treatment Decision-Making of Secondary Prevention After Venous Thromboembolism: Data From the Real-Life START2-POST-VTE Register. | No | Cancer patients |
| Predicting Recurrent Venous Thromboembolism in Patients With Deep-Vein Thrombosis: Development and Internal Validation of a Potential New Prediction Model (Continu-8). | No | Cancer patients |
| Risk factors for recurrence in deep vein thrombosis patients following a tailored anticoagulant treatment incorporating residual vein obstruction. | No | Cancer patients |
| D-Dimer Levels and Vitamin K Antagonist Therapy in Deep Vein Thrombosis of the Legs. | No | Cancer patients |
| OSA Is a Risk Factor for Recurrent VTE. | No | Cancer patients |
| Predictive Value of Mean Platelet Volume for Pulmonary Embolism Recurrence. | No | Cancer patients |
| Presence and degree of residual venous obstruction on serial duplex imaging is associated with increased risk of recurrence and progression of infrainguinal lower extremity deep venous thrombosis. | No | Cancer patients |
| Prospective study of natural history of deep vein thrombosis: early predictors of poor late outcomes. | No | Cancer patients |
| Management and outcomes of axial isolated distal deep vein thrombosis at North Shore Hospital, New Zealand: a retrospective audit. | No | Cancer patients |
| Predictive value of factor VIII levels for recurrent venous thrombosis: results from the MEGA follow-up study. | No | Cancer patients |
| D-dimer and residual vein obstruction as risk factors for recurrence during and after anticoagulation withdrawal in patients with a first episode of provoked deep-vein thrombosis. | No | Cancer patients |
| Predictive value of D-dimer test for recurrent venous thromboembolism at hospital discharge in patients with acute pulmonary embolism. | No | Cancer patients |
| [Acute-phase reactants and markers of inflammation in venous thromboembolic disease: correlation with clinical and evolution parameters]. | No | Cancer patients |
| Post-treatment residual thrombus increases the risk of recurrent deep vein thrombosis and mortality. | No | Cancer patients |
| Posttreatment ultrasound-detected residual venous thrombosis: a risk factor for recurrent venous thromboembolism and mortality. | No | Cancer patients |
| Does the location of thrombosis determine the risk of disease recurrence in patients with proximal deep vein thrombosis?. | No | Cancer patients |
| Evolution of deep venous thrombosis: a 2-year follow-up using duplex ultrasound scan and strain-gauge plethysmography. | No | Cancer patients |
| Risk of venous thromboembolism recurrence: high negative predictive value of D-dimer performed after oral anticoagulation is stopped. | No | Cancer patients |
| Predictors of residual venous obstruction after deep vein thrombosis of the lower limbs: a prospective cohort study. | No | Cancer patients |
| Prospective assessment of the natural history of positive D-dimer results in persons with acute venous thromboembolism (DVT or PE). | No | Cancer patients |
| Propagation, rethrombosis and new thrombus formation after acute deep venous thrombosis. | No | Cancer patients |
| D-Dimer Levels and Risk of Recurrent Venous Thromboembolism | No | Cancer patients |
| Tissue Factor As a Predictor of Recurrent Venous Thromboembolism in Malignancy: Biomarker Analyses of the CATCH Trial | No | Cancer patients |
| Normalization rates of compression ultrasonography in patients with a first episode of deep vein thrombosis of the lower limbs: association with recurrence and new thrombosis. | No | Cancer patients |
| Assessment of d-dimer value at first venous thromboembolism and risk of recurrence | No | unusual site thrombosis" |
| Elevated Gas6 plasma level as a predictor of venous thromboembolism recurrence and mortality in a prospective multicenter cohort of elderly patients with venous thromboembolism | No | Duplicated |
| Risk assessment model to predict recurrence in patients with unprovoked deep vein thrombosis or pulmonary embolism | No | Duplicated |
| External validation and updating of the Vienna Prediction Model for recurrent venous thromboembolism using a pooled individual patient data database | No | Duplicated |
| External validation of the dash prediction model: The trip (thrombosis research Italian partnership) collaboration | No | Duplicated |
| Predicting recurrent venous thromboembolism in patients with deep-vein thrombosis: External validation of a prediction model | No | Duplicated |
| The effect of an age-adjusted D-dimer on prediction of venous thromboembolism recurrence | No | Duplicated |
| Comorbidities, alone and in combination with D-dimer, as risk factors for recurrence after a first episode of unprovoked venous thromboembolism in the extended follow-up of the PROLONG study. | No | Duplicated |
| Risk factors of recurrent venous thromboembolism in patients with a first episode of unprovoked pulmonary embolism: Results from the PADIS PE multicenter, double-blind, randomized trial | No | Duplicated |
| Use of D-dimer testing to determine duration of anticoagulation, risk of cardiovascular events and occult cancer after a first episode of idiopathic venous thromboembolism: The extended follow-up of the PROLONG study | No | Duplicated |
| Patients with high levels of circulating endothelial progenitor cells (EPC) following at least three months of anticoagulation for unprovoked venous thromboembolism (VTE) are at low risk of recurrent VTE—Results from the ExACT randomised controlled trial. | No | Duplicated |
| Long-term risk of recurrent venous thromboembolism after a first contraceptive-related event: Data from REVERSE cohort study | No | Duplicated |
| Abnormal Protac-induced coagulation inhibition chromogenic assay results are associated with an increased risk of recurrent venous thromboembolism | No | Duplicated |
| D-dimer testing to determine duration of anticoagulation and risk of occult cancer after a first episode of idiopathic venous thromboembolism: the extended follow-up of the PROLONG study | No | Duplicated |
| D-dimer: A useful tool in gauging optimal duration of oral anticoagulant therapy? | No | Duplicated |
| D-dimer testing, with gender-specific cutoff levels, is of value to assess the individual risk of venous thromboembolic recurrence in non-elderly patients of both genders: a post hoc analysis of the DULCIS study. | No | Duplicated |
| The negative predictive value of D-dimer on the risk of recurrent venous thromboembolism in patients with multiple previous events: a prospective cohort study (the PROLONG PLUS study). | No | Duplicated |
| D-dimer, FVIII and thrombotic burden in the acute phase of deep vein thrombosis in relation to the risk of post-thrombotic syndrome | No | Recurrence was not studied |
| Management of Secondary Prevention in Venous Thromboembolism: Use of Reduced Doses of Rivaroxaban and Apixaban in Extended Therapy | No | Recurrence was not studied |
| D-dimer levels during and after anticoagulation withdrawal in patients with venous thromboembolism treated with non-vitamin K anticoagulants. | No | Recurrence was not studied |
| Does age-adjusted D-Dimer have a role in assessment of VTE recurrence rates?. | No | Recurrence was not studied |
| Assessment of coexisting deep vein thrombosis for risk stratification of acute pulmonary embolism. | No | Recurrence was not studied |
| "HERDOO2" clinical decision rule to guide duration of anticoagulation in women with unprovoked venous thromboembolism. Can I use any d-Dimer?. | No | Recurrence was not studied |
| Age and gender specific cut-off values to improve the performance of D-dimer assays to predict the risk of venous thromboembolism recurrence. | No | Recurrence was not studied |
| Circulating tissue factor positive microparticles in patients with acute recurrent deep venous thrombosis. | No | Recurrence was not studied |
| Deep venous thrombosis in patients with acute pulmonary embolism: prevalence, risk factors, and clinical significance. | No | Recurrence was not studied |
| D-dimer levels and 15-day outcome in acute pulmonary embolism. Findings from the RIETE Registry. | No | Recurrence was not studied |
| Markers of plasma coagulation and fibrinolysis after acute deep venous thrombosis. | No | Recurrence was not studied |
| Natural history of proximal deep vein thrombosis assessed by duplex ultrasound. | No | Recurrence was not studied |
| The post-thrombotic syndrome in young women: retrospective evaluation of prognostic factors. | No | Recurrence was not studied |
| [Prospective 12-year follow-up study of clinical and hemodynamic sequelae of deep venous thromboses in patients with low risk (Zurich Study)]. | No | Recurrence was not studied |
| Increased factor VIII levels in patients treated for venous thrombosis | No | Recurrence was not studied |
| The risk of recurrent venous thromboembolism in men and women. | No | Recurrence was not studied |
| Soluble p-selectin, D-dimer, and high-sensitivity C-reactive protein after acute deep vein thrombosis of the lower limb. | No | Recurrence was not studied |
| Prothrombotic plasma fibrin clot phenotype is predictive of recurrent venous thromboembolism following discontinuation of anticoagulant therapy | No | Recurrence was not studied |
| Prognostic value of elevated D-Dimer level after 1 month of anticoagulant therapy in patients with venous thromboembolism: Results of the 10-year follow-up | No | Recurrence was not studied |
| More positive D-dimer during anticoagulation with doacs and early after their discontinuation when compared with anticoagulation with vitamin K antagonist (VKA): A case-control study in patients with venous thromboembolism (VTE) | No | Recurrence was not studied |
| Repeated serial D-dimer (DD) measurement after anticoagulation therapy (AT) withdrawal to identify patients (PTS) at risk for venous thromboembolism (VTE) recurrence | No | Recurrence was not studied |
| Factors influencing the length of anticoagulant therapy in thromboembolic disease | No | Recurrence was not studied |
| Correlation between D-dimer and the persistence of residual thrombosis on ultrasound doppler at the end of anticoagulant treatment in deep vein thrombosis | No | Recurrence was not studied |
| High plasma levels of factor VIII and factor IX increased the incidence of recurrent VTE | No | Recurrence was not studied |
| Prediction of recurrent venous thromboembolism by the activated partial thromboplastin time | No | Recurrence was not studied |
| Relevance of age as a risk factor for recurrence after first venous thromboembolic event in women | No | D-dimer or ultrasound were not measured |
| Risk of recurrence of venous thromboembolism assessment following discontinuation of initial treatment of anticoagulation | No | D-dimer or ultrasound were not measured |
| Risk factors for recurrence after the first venous thromboembolic event in women | No | D-dimer or ultrasound were not measured |
| Measurement of fVIII levels over time in consecutive patients after deep vein thrombosis | No | D-dimer or ultrasound were not measured |
| Sex-specific risk factors for recurrent venous thromboembolism | No | D-dimer or ultrasound were not measured |
| High plasma levels of factor VIII and the risk of recurrent venous thromboembolism | No | D-dimer or ultrasound were not measured |
| Incidence of recurrent venous thromboembolism in relation to clinical and thrombophilic risk factors: prospective cohort study | No | D-dimer or ultrasound were not measured |
| An impaired protein S/TFPI pathway is a strong and independent risk factor for recurrent venous thromboembolism in patients with first unprovoked deep vein thrombosis | No | D-dimer or ultrasound were not measured |
| Low-Dose Aspirin for Preventing Recurrent Venous Thromboembolism | No | D-dimer or ultrasound were not measured |
| Dash score for prediction of recurrent venous thromboembolism: Updated long-term outcomes from a singlecentre | No | D-dimer or ultrasound were not measured |
| Identification of Patients with Unprovoked Venous Thromboembolism and a Low Risk of Recurrence Estimated By the Vienna Prediction Model: A Prospective Cohort Management Study | No | D-dimer or ultrasound were not measured |
| Long-term anticoagulation decisions in men over 50 years old following a first unprovoked venous thrombosis is not aided by the DASH score | No | D-dimer or ultrasound were not measured |
| Risk of recurrence after a first unprovoked venous thromboembolism: external validation of the Vienna Prediction Model with pooled individual patient data. | No | D-dimer or ultrasound were not measured |
| Utility of the dash score after unprovoked venous thromboembolism: Yesngle centre study and long-term patient outcomes | No | D-dimer or ultrasound were not measured |
| Predicting recurrence after unprovoked venous thromboembolism: Comparison of DASH, HERDOO2, and vienna score in the same cohort of patients | No | D-dimer or ultrasound were not measured |
| Optimal duration and dose intensity of the anticoagulation therapy with doacs in venous thromboembolism: Retrospective, cohort study real choice | No | D-dimer or ultrasound were not measured |
| Is there any clinical or biological difference that distinguishes recurrence from non-recurrence after idiopathic PE? | No | Anticoagulation for less than 3 months |
| Use of age adjusted d-dimer monitoring to assist the withdrawal of anticoagulation following unprovoked pulmonary embolism | No | Anticoagulation for less than 3 months |
| Risk for Recurrent Venous Thromboembolism in Patients With Subsegmental Pulmonary Embolism Managed Without Anticoagulation A Multicenter Prospective Cohort Study | No | Anticoagulation for less than 3 months |
| ExACT: Extended anticoagulation treatment for VTE: A randomised trial | No | Protocol |
| Trial Protocol: a randomised controlled trial of extended anticoagulation treatment versus routine anticoagulation treatment for the prevention of recurrent VTE and post thrombotic syndrome in patients being treated for a first episode of unprovoked VTE (The ExACT Study). | No | Protocol |
| Deep vein thrombosis resolution, recurrence and post-thrombotic syndrome: A prospective observational study protocol | No | Protocol |
| The diagnostic value of compression ultrasonography in patients with suspected recurrent deep vein thrombosis. | no | diagnostic |
| D-dimer can predict the risk of recurrent venous thromboembolism but not of superficial vein thrombosis after A first episode of idiopathic venous thromboembolism | No | Superficial vein trhombosis |
| Increased risk for recurrent thromboembolic events during the first three months in patients with superficial vein thrombosis treated with tinzaparin | No | Superficial vein trhombosis |
| D-dimer testing, thrombophilia screening and recurrences in patients with venous thromboembolism: A 6-year follow-up | No | Pending |
| Risk stratification of recurrent venous thromboembolism | No | Cancer patients |
| The use of the REVERSE study clinical prediction rule for risk stratification after initial anticoagulation results in decreased recurrences in patients with idiopathic venous thromboembolism | No | Duplicated |
| Negative D-dimer not sufficient to stop anticoagulation in men | No |  |
| Anticoagulation quality and the risk of recurrence of venous thromboembolism [3] | No |  |
| Presence of residual venous thrombus at warfarin withdrawal: A predictor for recurrence after a first episode of symptomatic provoked proximal deep venous thrombosis in an Asian population? | No |  |
| Evaluation of Venous Thromboembolism Recurrence Scores in an Unprovoked Pulmonary Embolism Population: A Post-hoc Analysis of the PADIS-PE trial | No |  |
| Factor XI and recurrent venous thrombosis: an observational cohort study. | No |  |
| D-dimer at venous thrombosis diagnosis is associated with risk of recurrence. | No |  |
| Validating the HERDOO2 rule to guide treatment duration for women with unprovoked venous thrombosis: multinational prospective cohort management study. | No |  |
| External validation of the DASH prediction rule: a retrospective cohort study. | No |  |
| D-dimer levels over time and the risk of recurrent venous thromboembolism: an update of the Vienna prediction model. | No |  |
| Different cut-off values of quantitative D-dimer methods to predict the risk of venous thromboembolism recurrence: a post-hoc analysis of the PROLONG study. | No |  |
| Prospective evaluation of the clinical deterioration in post-thrombotic limbs. | No |  |
| Yesx months versus two years of oral anticoagulation after a first episode of unprovoked deep-vein thrombosis. The PADIS-DVT randomized clinical trial | No |  |
