# Supplementary material for: D-dimer and lower limb ultrasound as prognostic factors for recurrent deep venous thrombosis and pulmonary embolism: A systematic review and meta-analysis
Source: PLoS One. 2026 May 15;21(5):e0340158. doi: 10.1371/journal.pone.0340158 (PMC13178970; doi:10.1371/journal.pone.0340158)
Supplement: S3 Table — (DOCX) [file pone.0340158.s003.docx]

**Question:** Residual vein thrombosis (RVT) and prognosis of recurrent venous thromboembolism after discontinuation of anticoagulant therapy

| **Certainty assessment** | | | | | | | **№ of patients** | | **Effect** | | **Certainty** | **Importance** |
| --- | --- | --- | --- | --- | --- | --- | --- | --- | --- | --- | --- | --- |
| **№ of studies** | **Study design** | **Risk of bias** | **Inconsistency** | **Indirectness** | **Imprecision** | **Other considerations** | **the presence of residual vein thrombosis (RVT)** | **absence of residual vein thrombosis** | **Relative (95% CI)** | **Absolute (95% CI)** |  |  |
| **Rate of tromboembolic recurrence after stop anticoagulation (follow-up: range 6 months to 128 months)** | | | | | | | | | | | | |
| 10 | non-randomised studies | serious^a^ | serious^b^ | not serious | serious^c^ | none | 252/1431 (17.6%) | 184/1486 (12.4%) | **OR 2.00** (1.02 to 3.94) | **10 more per 100** (from 0 fewer to 23 more) | ⨁◯◯◯ Very low^a,b,c^ | CRITICAL |

**CI:** confidence interval; **OR:** odds ratio

#### Explanations

a. We downgraded the certainty of the evidence by one level because of serious risk of bias concerns. All studies had at least one QUIPS domain rated as high risk of bias or two domains rated as moderate risk of bias.

b. We downgraded the certainty of the evidence by one level because the prediction interval ranged from 0.19 to 21.26.

c. We downgraded the certainty of the evidence by one level because the confidence interval showed a small association at 1.02 and a large effect at 3.94.
