# Supplementary figures and images for: D-dimer and lower limb ultrasound as prognostic factors for recurrent deep venous thrombosis and pulmonary embolism: A systematic review and meta-analysis
Source: PLoS One. 2026 May 15;21(5):e0340158. doi: 10.1371/journal.pone.0340158 (PMC13178970; doi:10.1371/journal.pone.0340158)

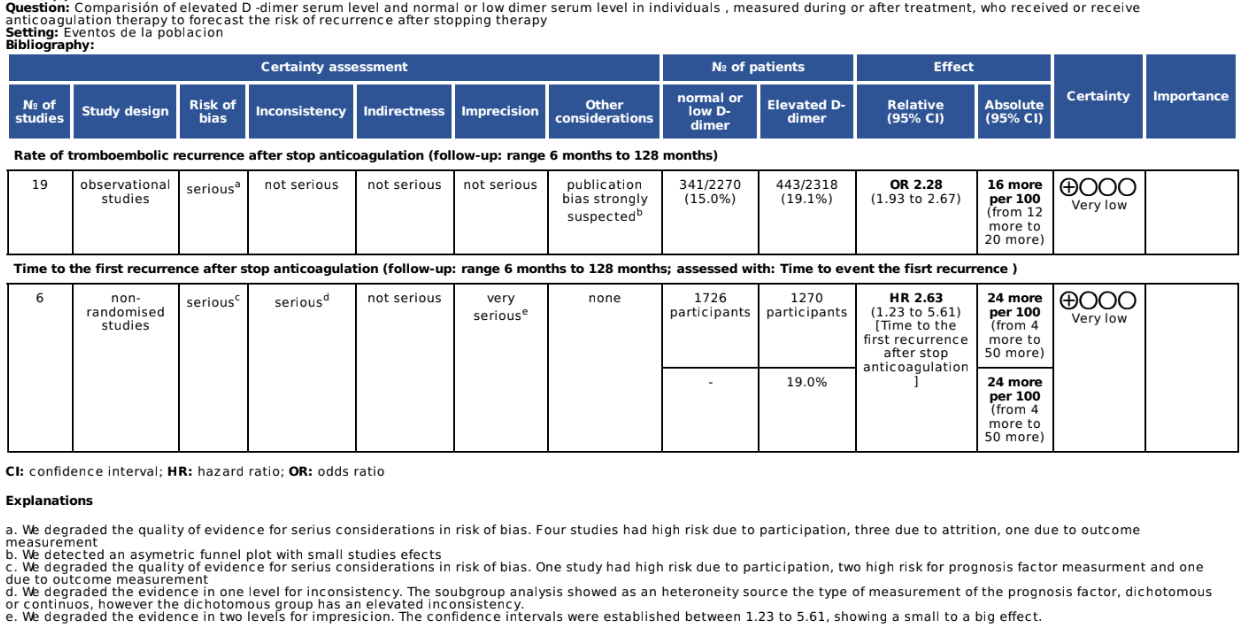

Supplement: S4 Table — (DOCX) [file pone.0340158.s004.docx]

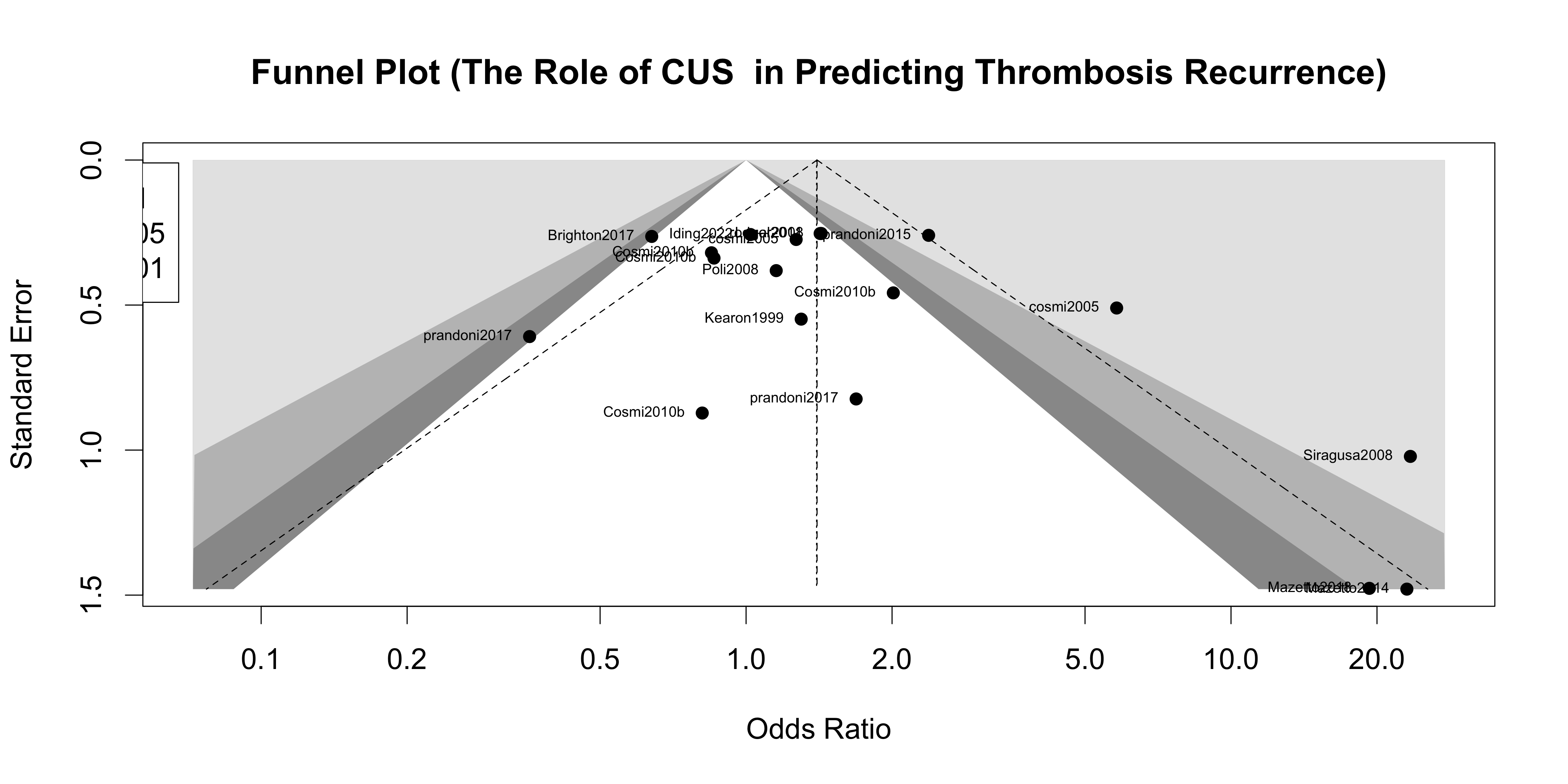

Supplement: S11 Fig — (TIFF) [file pone.0340158.s015.tiff]

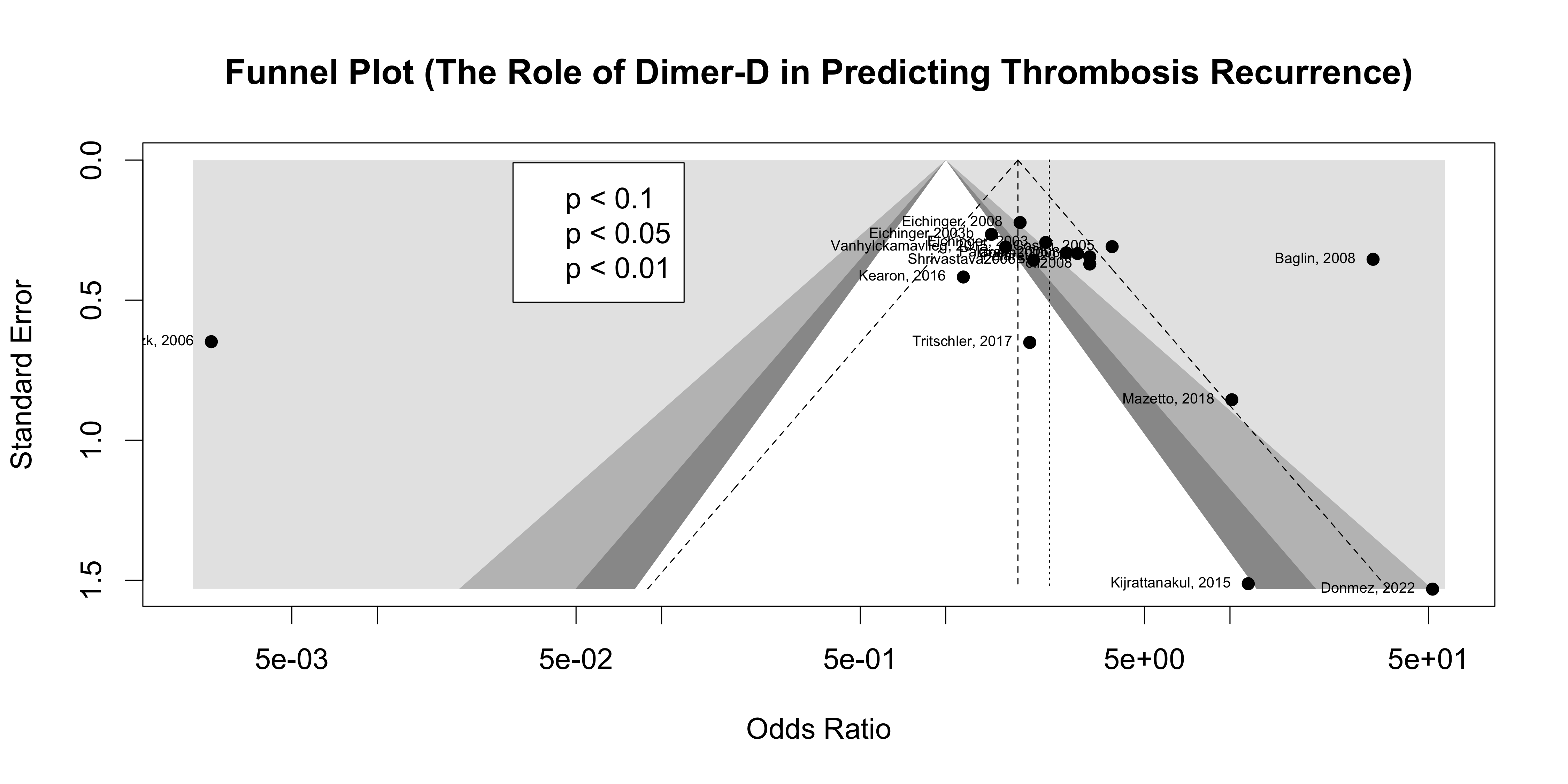

Supplement: S12 Fig — (TIFF) [file pone.0340158.s016.tiff]
